# Drought Influences Fungal Community Dynamics in the Grapevine Rhizosphere and Root Microbiome

**DOI:** 10.3390/jof7090686

**Published:** 2021-08-25

**Authors:** María Julia Carbone, Sandra Alaniz, Pedro Mondino, Matías Gelabert, Ales Eichmeier, Dorota Tekielska, Rebeca Bujanda, David Gramaje

**Affiliations:** 1 Departamento de Protección Vegetal, Facultad de Agronomía, Universidad de la República, 12900 Montevideo, Uruguay; mjcarbone@fagro.edu.uy (M.J.C.); salaniz@fagro.edu.uy (S.A.); pmond@fagro.edu.uy (P.M.); mgelatgut@gmail.com (M.G.); 2 Faculty of Horticulture, Mendeleum—Institute of Genetics, Mendel University in Brno, 69144 Lednice, Czech Republic; ales.eichmeier@mendelu.cz (A.E.); dorota.tekielska@mendelu.cz (D.T.); 3 Instituto de Ciencias de la Vid y del Vino (ICVV), Consejo Superior de Investigaciones Científicas—Universidad de la Rioja—Gobierno de La Rioja, 26071 Logroño, Spain; rebeca.bujanda@icvv.es

**Keywords:** black-foot disease, drought, high-throughput next generation sequencing, *Vitis vinifera* L., water stress

## Abstract

Plant roots support complex microbial communities that can influence nutrition, plant growth, and health. In grapevine, little is known about the impact of abiotic stresses on the belowground microbiome. In this study, we examined the drought-induced shifts in fungal composition in the root endosphere, the rhizosphere and bulk soil by internal transcribed spacer (ITS) high-throughput amplicon sequencing (HTAS). We imposed three irrigation regimes (100%, 50%, and 25% of the field capacity) to one-year old grapevine rootstock plants cv. SO_4_ when plants had developed 2–3 roots. Root endosphere, rhizosphere, and bulk soil samples were collected 6- and 12-months post-plantation. Drought significantly modified the overall fungal composition of all three compartments, with the root endosphere compartment showing the greatest divergence from well-watered control (100%). The overall response of the fungal microbiota associated with black-foot disease (*Dactylonectria* and “*Cylindrocarpon*” genera) and the potential biocontrol agent *Trichoderma* to drought stress was consistent across compartments, namely that their relative abundances were significantly higher at 50–100% than at 25% irrigation regime. We identified a significant enrichment in several fungal genera such as the arbuscular mycorrhizal fungus *Funneliformis* during drought at 25% watering regime within the roots. Our results reveal that drought stress, in addition to its well-characterized effects on plant physiology, also results in the restructuring of grapevine root microbial communities, and suggest the possibility that members of the altered grapevine microbiota might contribute to plant survival under extreme environmental conditions.

## 1. Introduction

Drought is one of the major environmental stresses in agriculture, resulting in significant economic losses worldwide [1]. Climate projections indicate that frequency and severity of drought events are likely to increase in some regions, which will require major adaptations in order to maintain agricultural production [1,2].

Mechanisms of water stress responses in plants involve adaptations at the morphological, physiological, and molecular levels [3]. Among those, drought avoidance (e.g., increased root growth, leaf rolling, and stomatal closure) and drought tolerance (e.g., osmotic adjustment, antioxidant defense system, and increased ABA production) are considered the two major general mechanisms to water stress resistance in plants [3,4].

Plants are closely related with microorganisms that inhabit in the soil-rhizosphere-root endosphere continuum [5,6]. These microorganisms can provide benefits to plants, contributing with nutrient mobilization and transport, protection against pathogens or pests, and stress alleviation [6,7,8].

Changes in environmental factors that affect plants are also expected to influence plant-associated microbiomes, and vice versa [9]. Drought affects the structure of microbial soil community [10,11] as a result of the selection in favor of taxa tolerant to low moisture content and shifts in diffusion rates and soil chemistry [12]. Edaphic communities are the predominant source of microorganisms for the root-associated compartments, therefore changes in the microbial soil community caused by drought have consequences on the composition of root-associated microbiome [5]. Additionally, plant responses to drought stress, such as modifications in the root morphology [13] and shifts in root exudation profile [14,15], also directly affect the activity and structure of root-associated microbial communities [5,12].

Many studies have noticed the influence of drought on root-associated bacterial communities. Bouasria et al. [16] observed that drought had a significant effect on rhizosphere bacterial community diversity in different species of grass. Barnard et al. [10] studied the responses of soil bacterial and fungal communities to desiccation and rewetting in grasslands and concluded that only bacterial communities were significantly affected by dry-down. An enrichment of bacterial taxa *Actinobacteria* in the root endosphere and rhizosphere of drought-treated plants, as well as in the soil, has been observed across multiple host species [5,17,18,19,20,21].

In contrast, the effect of drought on soil and root-associated fungal communities remains largely unexplored [10,12,16,17,22]. Barnard et al. [10] observed that soil fungal communities in grasslands were largely unaffected by dry-down, suggesting a high degree of resistance to changes in soil water availability. Similar results were found by Naylor et al. [17], who observed that drought had no significant effect on soil, rhizosphere, and root endosphere fungal community composition in various grass lineages. However, recent research demonstrated that drought significantly altered the rhizosphere and root endosphere fungal community diversity on rice crop plant [5].

Grapevine (*Vitis vinifera* L.) is a traditionally non-irrigated crop [23]. Nevertheless, agronomic practices such as rootstock use, tillage or no-tillage, controlled cover crop, and irrigation are largely used to balance vine vegetative and reproductive growth [23,24]. In the context of predicted increased drought events, management strategies including use of drought-resistant cultivars [25] and soil conservation measures [24] are being increasingly employed.

The belowground grapevine microbiome is affected in composition and diversity by soil-plant compartment [26,27]. Diversity of bacterial [26] and fungal [27] communities is higher in the rhizosphere and the bulk soil compared with the root endosphere compartment. Plant-associated properties such as rootstock genotype [28,29] and phenological stage [30] play a significant role in shaping grapevine microbiome. Environmental factors related with soil physicochemical properties and moisture content [26,31,32,33], as well as management practices such as tillage and irrigation [24], has been identified as factors that significantly influence in the grapevine rhizosphere microbiome diversity.

Potential black-foot disease (BFD) pathogens have been found determining the dissimilarities in the fungal microbiome between soil-grapevine compartments [27]. BFD of grapevine is an important disease in nurseries and young vineyards in most grapevine growing regions worldwide [34,35]. Symptoms include necrotic lesions on root tissue and black discoloration and necrosis of wood tissue in the base of the rootstock, which lead to the death of young vines [34]. Causal agents of BFD are soilborne pathogens belonging to genera *Campylocarpon*, *Cylindrocladiella*, *Dactylonectria*, *Ilyonectria*, *Neonectria, Pleiocarpon*, and *Thelonectria* [36,37]. BFD incidence is favored by poor drained conditions, high moisture content, and heavy texture of soil [34].

Grapevine physiological and molecular responses to water stress has been extensively studied [23]; however, little is known about the effect of drought on fungal microbiome structure and composition in the bulk soil, rhizosphere, and root endosphere in grapevine. Understanding the effect of extreme environments on fungal community composition will provide information about how the network of grapevine-microbiome interactions is reshaped under challenging scenarios. Moreover, identification of root-associated fungal taxa that develop under drought conditions could lead to the detection of beneficial fungal symbionts that are able to mediate plant stress tolerance through diverse mechanisms.

In this study, we conducted a greenhouse-based experiment to explore the impact of drought on the root-associated fungal communities of cultivated grapevine. The compositional shifts in the bulk soil, rhizosphere, and root endosphere communities in the most prevalent grapevine rootstock genotype and predominant soil type of the main grapevine growing area in Uruguay were examined. In addition, the effect of drought on the metabolic function of the fungal communities in the three plant compartments was analyzed. We were particularly interested in evaluating the impact of extreme environments on BFD fungal abundance. This approach has allowed us to determine the conservation and extent of the drought-mediated shifts accomplished by the beneficial and pathogenic fungal microbiota.

## 2. Materials and Methods

### 2.1. Experimental Design and Treatments

In November 2018, seventy-two Selection Oppenheim 4 (SO4; *Vitis berlandieri X Vitis riparia*) rootstock cuttings, previously callused, were planted in 11 L pots (one plant per pot) containing natural soil (Table S1). The soil was collected from a commercial grapevine nursery (34°34′48″ S, 56°17′50″ W) located in Canelones, the major grape-growing region in Uruguay [38]. During the experiment, the potted plants were maintained in a greenhouse located in the Faculty of Agronomy (Montevideo, Uruguay). Rootstock SO4 is the most widely used rootstock in Uruguay, accounting for 63% of the grapevine growing area [39].

One month after sprouting, potted SO4 plants were randomly divided into three treatments, simulating three types of irrigation regimes. The treatments were (1) irrigation at 25% of field capacity (severe water deficit: SWD), (2) irrigation at 50% of field capacity (moderate water deficit: MWD), and (3) irrigation at 100% of field capacity (absence of water deficit: AWD). Soil water content at field capacity was previously calculated according to Silva et al. [40]. An automated drip irrigation system was adjusted for each irrigation treatment, by measuring the dielectric constant of soil. The irrigation treatments were maintained over 18 months. The experimental design consisted of four randomized blocks per irrigation treatment, each containing 6 plants (24 plants per irrigation treatment).

During the growing season, predawn leaf water potential was measured every 20 days approximately with a pressure chamber [41]. After the leaf fall, 18 months from the beginning of the experiment (July 2020), plants were pruned, and the pruning weight was registered. The data were subjected to analysis of variance and mean values were separated according to Tukey’s honestly significant difference at *p*-value = 0.05, with Statistix 10 software (Analytical Software, Tallahasese, FL, USA).

### 2.2. Sample Collection

Bulk soil, rhizosphere (soil surrounding roots), and root samples were collected at six months (June 2019) and twelve months (December 2019) after the irrigation treatments were established. Twenty-four replicates were collected from each plant compartment and irrigation treatment.

Bulk soil samples were collected with a sterile shovel close to the edge of the pot at depths of 15–20 cm. Roots and rhizosphere were collected with a sterile spade close to the stem at depths where the root system was denser [27]. Samples were kept in sterile bags and stored on dry ice at the time of sampling. Immediately, samples were transported to the laboratory for further processing. Roots with rhizosphere particles attached were separated according to Berlanas et al. [29]. After that, roots were rinsed and cleaned with distilled water and surface disinfected with sodium hypochlorite (1%) for 30 s. Finally, roots were washed three times with sterile distilled water and the root cortex was removed with sterile scalpel. All samples were stored at −80 °C until DNA extraction. A total of 432 samples were collected.

### 2.3. DNA Extraction, Amplification and Sequencing

Genomic rhizosphere and bulk soil DNA were extracted from 0.5 g sample using the DNeasy PowerSoil Kit (Qiagen, Hilden, Germany), following the kit protocol. The root endosphere DNA was extracted from 0.05 g sample using the DNeasy Plant Pro Kit (Qiagen, Hilden, Germany). Roots were first ground into powder in liquid nitrogen and then the kit protocol was followed. DNA yields were quantified using the Invitrogen Qubit 4 Fluorometer with Qubit dsDNA HS (High Sensitivity) Kit (Thermo Fisher Scientific, Waltham, MA, USA) and the extracts were adjusted to 10–15 ng/μL. After DNA quantification, samples were pooled in groups of two, resulting in a total of twelve replicates per plant-compartment, irrigation treatment, and sampling time for every batch of 24 plants.

For fungal library preparation, the complete fungal ITS2 region was amplified using the primers ITS86F [42] and ITS4 [43]. Primers were modified to include the Illumina sequencing primers. PCR were carried out in a final volume of 25 μL, containing 2.5 μL of template DNA, 0.5 μM of the primers, 12.5 μL of Supreme NZYTaq 2× Green Master Mix (NZYTech), and ultrapure water up to 25 μL. PCR amplifications consisted of an initial denaturation step at 95 °C for 5 min, followed by 35 cycles of 95 °C for 30 s, 49 °C for 30 s, 72 °C for 30 s, and a final extension step at 72 °C for 10 min. A secondary PCR was conducted to index the amplicons with identical conditions, but for only 5 cycles and with 60 °C as the annealing temperature. Libraries were purified using the Mag-Bind RXNPure Plus magnetic beads (Omega Biotek, Norcross, GE, USA), following the instructions provided by the manufacturer. The purified libraries were pooled in equimolar amounts according to the quantification data provided by the Qubit dsDNA HS Assay (Thermo Fisher Scientific, Waltham, MA, USA). Samples were sequenced in the MiSeq platform (Illumina, San Diego, CA, USA) at the AllGenetics and Biology SL (Galicia, Spain) (www.allgenetics.eu, accessed on 15 May 2020, using a paired-end 2 × 300 bp (PE 300) sequencing and the MiSeq Reagent Kit v3 (Illumina, San Diego, CA, USA). Negative controls during library preparation and DNA extraction, and a positive control containing DNA of a grapevine rhizosphere sample [29] were included.

### 2.4. Data Analysis

Sequence quality was visualized using FastQC-0.10.1 [44]. The further data processing was completed using SEED v2.0 [45]. Raw forward and reverse sequences for each sample were assembled into paired-end reads using the fastq-join 1.1.2 tool from the eatools suite [46]. Sequences were then quality filtered, Q = 30; trimmed on the length > 250 bases; and ambiguous bases were removed. Sequences were grouped by barcode motives and then labelled by sample names. Fungal ITS were extracted using ITSx 1.0.11 [47]. Then, the sequences were clustered into operational taxonomic units (OTUs) and chimeric sequences were removed with Usearch-UPARSE 8.1.1861 [48] with a threshold of 97% pairwise identity against UNITE fungal dynamic database [49]. The representative consensus sequences were extracted from the clusters using MAFFT 7.222 [50]. Finally, the identification of OTUs was performed by blastn, tblastx, and makeblastdb 2.5.0+ (https://blast.ncbi.nlm.nih.gov/Blast.cgi, accessed on 28 September 2020). The dataset was normalized applying Total Sum Scaling standard approach and samples were rarefied to 15,736 fungal sequences (the minimum library size).

### 2.5. Fungal Diversity and Statistical Analysis

Alpha diversity was calculated using Shannon and Chao1 indices in Phyloseq package, as implemented in MicrobiomeAnalyst [51,52]. Beta diversity was estimated using a principal coordinates analysis (PCoA) based on Bray–Curtis metrics [53] with MicrobiomeAnalyst. PERMANOVA analysis was carried out to evaluate which OTUs significantly differed in abundance among experimental factors. Good’s coverage values and rarefaction curves were calculated using MicrobiomeAnalyst.

The Linear Discriminant Analysis Effect Size (LEfSe) algorithm was used to identify taxa (genus level or higher) that differed in relative abundance between water stress regime and time of sampling among each compartment [54]. MicrobiomeAnalyst LEfSe implementation was used; the threshold for the logarithmic Linear Discriminant Analysis (LDA) score was set at 2.0 and the FDR-adjusted *p*-value cutoff at 0.1. The fungal OTUs shared among compartments, water stress regimes and time of sampling were obtained by a Venn diagram analysis using the software available at http://bioinformatics.psb.ugent.be, accessed on 15 October 2020. Correlation network analysis was performed by MicrobiomeAnalyst based on the SparCC algorithm [55]. The permutation was settled at 100 with a *p*-value threshold of 0.01 and a correlation threshold of 0.5 at the genus taxonomical level.

### 2.6. Functional Prediction of Fungal Communities

The function of fungal communities in the three irrigation conditions in the soil-plant compartments was investigated using FUNGuild v1.0 [56]. According to three trophic modes (pathotrophs, saprotrophs, and symbiotrophs), eleven guilds were classified: plant pathogens, animal pathogens, fungal parasites, lichen parasites, undefined saprotrophs, soil saprotrophs, wood saprotrophs, dung saprotrophs, plant saprotrophs, endophytes, and arbuscular mycorrhizal. OTUs that did not match taxa in the database were classified as “unassigned”. Guilds considered “probable” and “highly probable” according to the fungal database were selected for further analysis. Relative abundance of OTUs according to guilds were calculated to the three irrigation conditions at the three soil-plant compartments analyzed. The effect of water stress conditions on the relative abundance of OTUs according to the trophic modes was assessed performing ANOVA, with Statistix 10 software (Analytical Software). Data were transformed to $\sqrt{x}$ prior to analysis. Transformed data means were compared using Tukey’s honestly significant difference at *p*-value = 0.05.

## 3. Results

### 3.1. Water Potential and Pruning Weight

Predawn water potential and the pruning weight data differed among irrigation treatments (*p* < 0.05). In the 2018–2019 growing season, the predawn water potential ranged from −0.70 to −0.60 MPa in the SWD regime, from −0.45 to −0.36 MPa in the MWD regime, and from −0.12 to −0.09 MPa in the AWD regime. In the 2019–2020 growing season, the predawn water potential ranged from −0.78 to −0.58 MPa, from −0.51 to −0.28 MPa, and from −0.14 to −0.07 in the SWD, MWD, and AWD regimes, respectively (Figure S1). The pruning weight in the SWD regime was significantly lower (1.72 g/plant) compared to the MWD (31.25 g/plant) and the AWD (54.83 g/plant) regimes (Figure S2).

### 3.2. High-Throughput Amplicon Sequencing

A total of 4,963,651 fungal ITS sequences were generated from 207 samples after paired-end alignments, quality filtering, and deletion of singletons, chimeric, chloroplast, and mitochondrial sequences. Nine samples were removed from the analysis due to the low number of sequences reads. Sequences were assigned to 339 fungal OTUs (Table S2). Good’s coverage values in all samples ranged from 99.9 to 100% (Table S3). Chao1 diversity estimator ranged from 15.0 to 87.5, while Shannon diversity estimator ranged from 1.7 to 3.2 (Table S3). Sequencing data are deposited under BioProject acc. No. PRJNA707008, where the SRA experiments are available by acc. Nos. SRX10263838–SRX10366007.

### 3.3. Fungal Communities Differed among Soil-Plant Compartments

**Figure 1:**
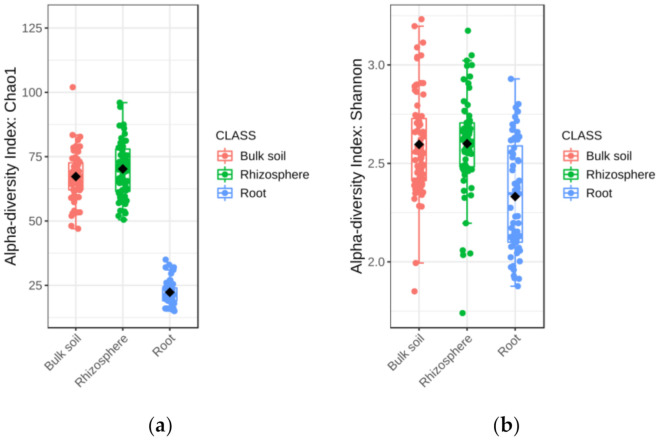
Boxplot illustrating the differences in (**a**) Chao1 and (**b**) Shannon diversity measures of the fungal communities in the soil-plant compartments.

The alpha diversity of fungal communities differed significantly among soil-plant compartments (Table 1). Chao1 and Shannon’s estimators indicated that fungal diversity in rhizosphere and bulk soil was significantly higher than in root (Figure 1a,b). Principal coordinates analysis (PCoA) of Bray–Curtis data demonstrated that soil-plant compartment was a source of beta diversity (*R*^2^ = 0.15, *p* < 0.001) (Figure S3). The relative abundance of fungal phylum, family, and genus detected in bulk soil, rhizosphere, and root is shown in Figure S4. Considering data from all soil-plant compartments, the most abundant phyla were Ascomycota, followed by Basidiomycota, and Mucoromycota (Figure S4a).

In the root, the most abundant families were Nectriaceae (18.5%), Ceratobasidiaceae (12.2%), and Mortierellaceae (7.8%) (Figure S4b). In the rhizosphere, most abundant families were Mortierellaceae (18.7%), followed by Nectriaceae (16.4%), and Ceratobasidiaceae (9.9%), whereas in bulk soil, the most abundant families were Mortierellaceae (25.2%), followed by Nectriaceae (13.8%), and an unidentified family (8.2%) (Figure S4b).

### 3.4. Sampling Time Influence on Fungal Diversity

Fungal microbiome diversity significantly differed between sampling times in the root and bulk soil (Table 1). In root samples, Shannon diversity increased towards the 12-month sampling, whereas the opposite was found in bulk soil samples (Figure S5). The Bray–Curtis metric of beta diversity also was affected by sampling time in root (*R*^2^ = 0.22, *p* < 0.001) and bulk soil (*R*^2^ = 0.24, *p* < 0.001), which reinforced the differences observed in the community composition over time (Figure S6a,c). In the rhizosphere, sampling time did not predict alpha diversity (Table 1), but affected the Bray–Curtis metrics of beta diversity (*R*^2^ = 0.30, *p* < 0.001) (Figure S6b).

Regarding fungal OTUs, the proportion of OTUs shared among both sampling times was 33.2% in the root compartment (Figure S7a), 74.5% in the rhizosphere (Figure S7b), and 68.6% in bulk soil (Figure S7c).

The LEfSe detected that 26 genera determined the dissimilarities among sampling times in root (Figure S8a). In the rhizosphere, the relative abundance of 56 genera was affected by sampling time, whereas 45 genera determined the dissimilarities between sampling times in bulk soil (Figure S8b,c).

### 3.5. Water Deficit Affects Fungal Diversity in Soil-Plant Compartments

Our results demonstrated that fungal microbiome varied significantly among irrigation regimes. This pattern was consistent to community-level measure of alpha diversity in root, rhizosphere, and bulk soil in both sampling times (Table 1). The relative abundance of fungal phyla, family, and genus detected across the soil-plant compartments in the different conditions of water stress is shown in Figure S9.

**Figure 2:**
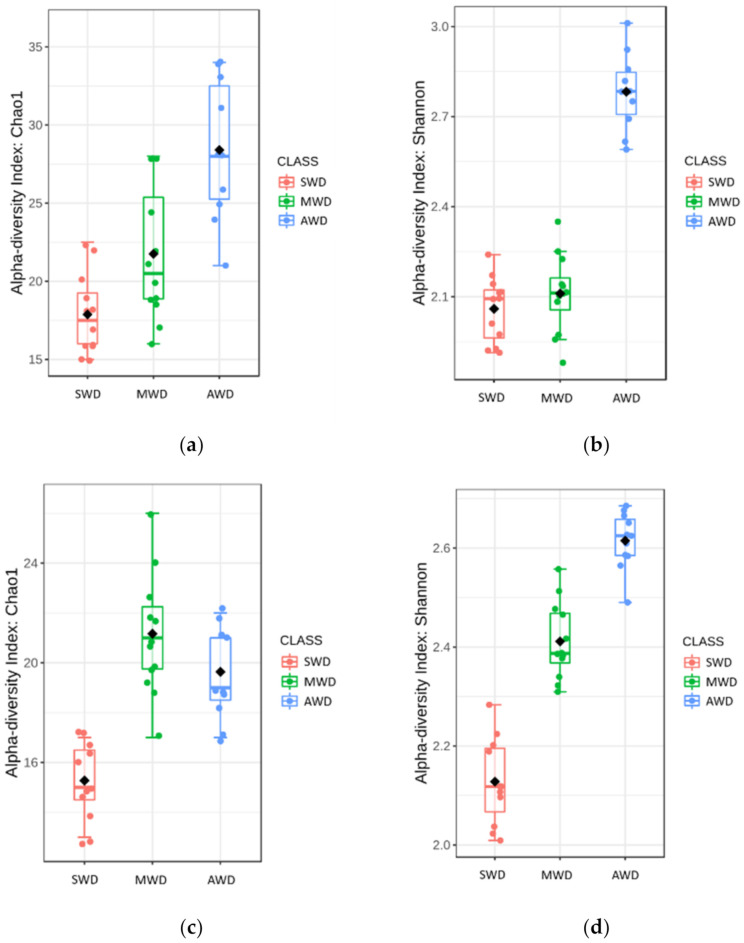
Boxplot illustrating the differences in (**a**) Chao1 and (**b**) Shannon diversity measures at the 6-month sampling time and (**c**) Chao1 and (**d**) Shannon diversity measures at the 12-month sampling time of the fungal communities in the root, at dif-ferent irrigation regimes: severe water deficit (SWD), moderate water deficit (MWD), and absence of water deficit (AWD).

**Figure 3:**
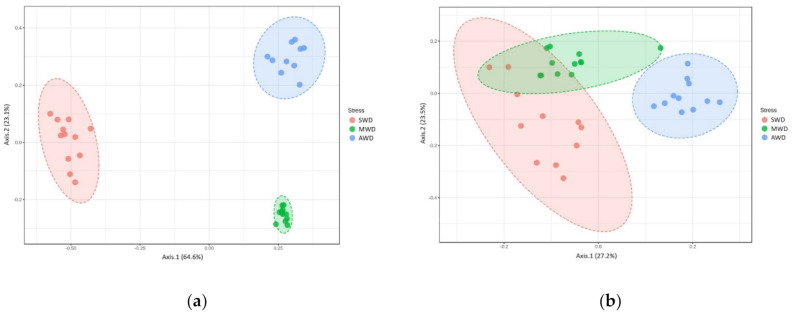
Principal Coordinate Analysis (PCoA) based on Bray–Curtis dissimilarity metrics, showing the distance in the fungal communities among irrigation regimes (SWD: severe water deficit, MWD: moderate water deficit, and AWD: absence of water deficit) at (**a**) 6-month sampling and (**b**) 12-month sampling times in the root.

Regarding root samples, the richness and diversity of OTUs in the AWD regime (Sampling time 1 = Chao1: 28.40 ± 1.42, Shannon: 2.78 ± 0.04; Sampling time 2 = Chao1: 19.64 ± 0.56, Shannon: 2.61 ± 0.02) was significantly higher than in the SWD regime (Sampling time 1 = Chao1: 17.88 ± 0.74, Shannon: 2.06 ± 0.03; Sampling time 2 = Chao1: 15.27 ± 0.45, Shannon: 2.13 ± 0.03) in both sampling times (Figure 2). At 6-month sampling, the alpha diversity indexes of fungal communities were similar between SWD and MWD (Chao1: 21.75 ± 1.26; Shannon: 2.11 ± 0.04) regimes (Figure 2a,b). However, at the 12-month sampling time, alpha diversity measure in MWD regime (Chao1: 21.17 ± 0.71; Shannon: 2.41 ± 0.02) increased with respect to SWD treatment (Figure 2c,d). PCoA of Bray–Curtis data demonstrated that irrigation regime was a source of beta diversity at the 6-month (*R*^2^ = 0.86, *p* < 0.001) (Figure 3a) and at 12-month sampling times (*R*^2^ = 0.39, *p* < 0.001) (Figure 3b).

**Figure 4:**
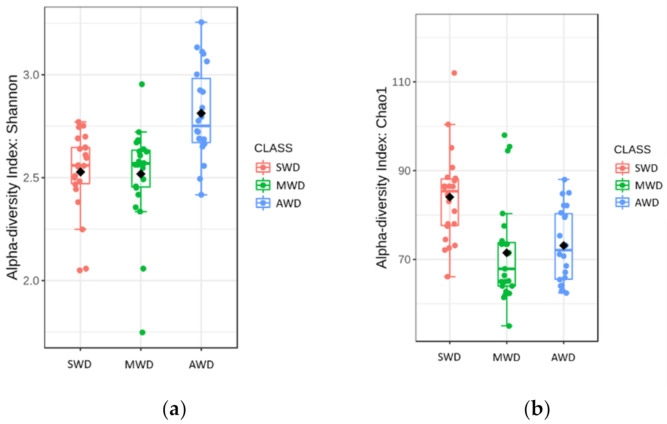
Boxplot illustrating the differences in (**a**) Chao1 and (**b**) Shannon diversity measures at both sam-pling times of the fungal communities in the rhizosphere at different irrigation regimes: severe water deficit (SWD), moderate water deficit (MWD), and absence of water deficit (AWD).

**Figure 5:**
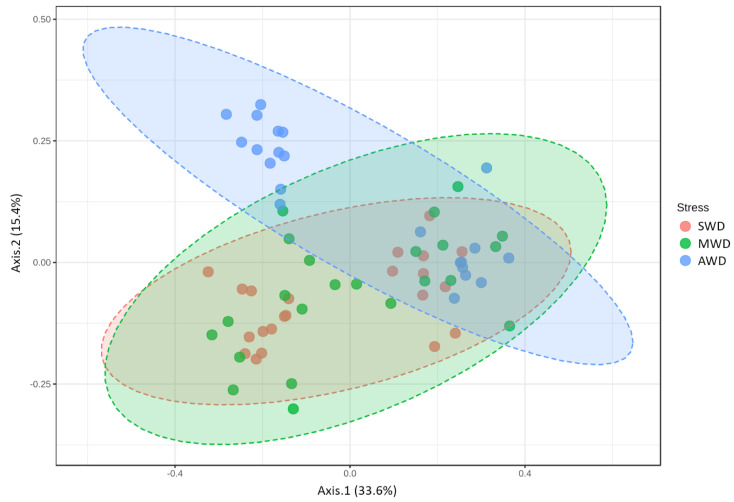
Principal Coordinate Analysis (PCoA) based on Bray–Curtis dissimilarity metrics, showing the distance in the fungal communities among irrigation regimes (SWD: severe water deficit, MWD: moderate water deficit, and AWD: absence of water deficit), at both sampling times of the fungal communities in the rhizosphere.

Rhizosphere samples at both sampling times were analzyed together, due to the lack of significant differences in the alpha diversity measures between sampling times (Table 1). Chao1 richness and Shannon diversity measures were affected by the irrigation regime, although there was not a clear pattern (Figure 4). Chao1 estimator measures were higher in SWD regime (84.04 ± 2.19) than in MWD (71.48 ± 2.37) and AWD (73.12 ± 1.78) regimes, whereas Shannon diversity estimator predicted the highest values in the AWD regime (2.81 ± 0.05) (Figure 4a,b). Bray–Curtis metric of beta diversity was affected by the irrigation regime (*R*^2^ = 0.12, *p* < 0.001) (Figure 5).

**Figure 6:**
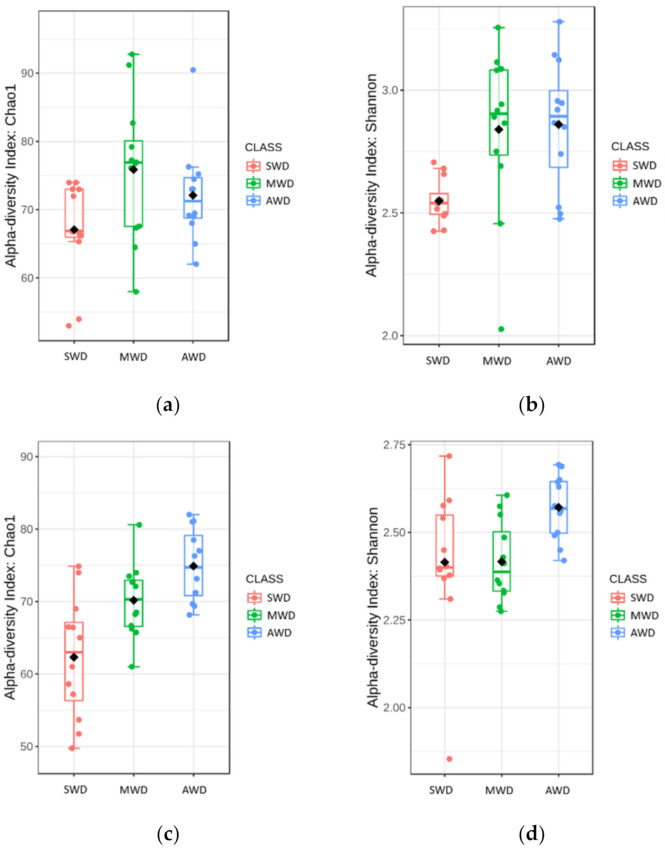
Boxplot illustrating the differences in (**a**) Chao1 and (**b**) Shannon diversity measures at 6-month sampling time and (**c**) Chao1 and (**d**) Shannon diversity measures at 12-month sampling time of the fungal communities in the bulk soil, at different irrigation regimes: severe water deficit (SWD), moderate water deficit (MWD), and absence of water deficit (AWD).

**Figure 7:**
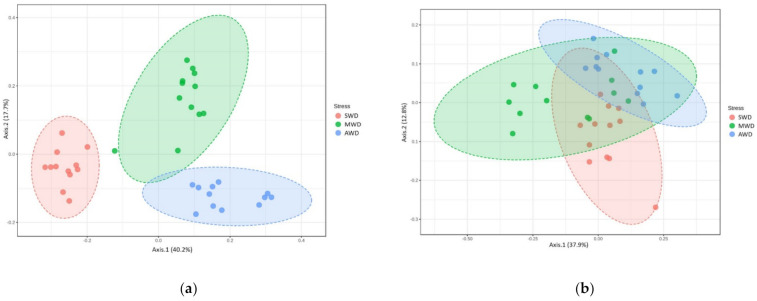
Principal Coordinate Analysis (PCoA) based on Bray–Curtis dissimilarity metrics, showing the distance in the fungal communities among irrigation regimes (SWD: severe water deficit, MWD: moderate water deficit, and AWD: absence of water deficit) at (**a**) 6-month and (**b**) 12-month sampling times in the bulk soil.

In the bulk soil at 6-month sampling, richness and diversity of OTUs in the MWD regime (Chao1: 75.87 ± 2.98; Shannon: 2.84 ± 0.10) was higher than in the SWD regime (Chao1: 67.06 ± 2.07; Shannon: 2.55 ± 0.03), but similar with the AWD regime (Chao1: 72.10 ± 2.07; Shannon: 2.86 ± 0.08) (Figure 6a,b). At 12-month sampling time, Chao1 diversity estimator indicated that the diversity in the AWD (74.89 ± 1.45) and MWD (70.17 ± 1.48) regimes was higher than in the SWD regime (62.32 ± 2.41), whereas Shannon estimator did not detect differences between SWD (2.41 ± 0.06) and MWD (2.42 ± 0.03) regimes, which were lower than the AWD (2.57 ± 0.03) (Figure 6c,d). Irrigation regimes also affected the Bray–Curtis metric of beta diversity at both 6-month (*R*^2^ = 0.52, *p* < 0.001) (Figure 7a) and 12-month sampling times (R^2^ = 0.25, *p* < 0.001) (Figure 7b).

Regarding the LEfSe analysis, 50 genera determined the dissimilarities in the fungal community among irrigation regimes in the root at 6-months sampling time, whereas 20 genera discriminated among irrigation regimes at 12-month sampling time (Figure S10a). Relative abundances of the black-foot fungi “*Cylindrocarpon*” and *Dactylonectria* significantly increased at AWD and MWD regimes, respectively, at both sampling times. *Thelonectria* spp. were more abundant at MWD at both sampling times. The fungal genus *Trichoderma*, a potential biocontrol agent of black-foot pathogens, was found with highest abundance at AWD treatment at 6-months sampling and at MWD treatment at 12-months sampling, and with the lowest abundance at SWD treatment at both sampling times. The genus *Funneliformis*, which is an arbuscular mycorrhizal fungus (AMF), showed significantly higher abundance at SWD than the other treatments at 6-month sampling time.

In the rhizosphere, the relative abundance of 51 genera determined the differences among irrigation regimes (considering both sampling time) (Figure S10b). Relative abundances of “*Cylindrocarpon*” and *Dactylonectria* significantly increased at AWD and MWD treatments, respectively. The fungal genus *Funneliformis* showed significantly higher abundance at SWD treatment than the other treatments.

In the bulk soil, the LEfSe detected 48 genera and 22 genera which determined the dissimilarities in the fungal community among irrigation regimes at 6-month and 12-month sampling times, respectively (Figure S10c). Relative abundance of “*Cylindrocarpon*” and *Trichoderma* were significantly higher at MWD treatment than the other treatments at 6-month sampling time. *Thelonectria* relative abundance was higher at AWD at 6-months sampling time and at MWD at 12-month sampling time. *Funneliformis* showed significantly higher abundance at SWD treatment than the other treatments, at 6-month sampling time, although its abundance was higher at AWD treatment than the other treatments at 12-month sampling time.

### 3.6. Irrigation Regime-Specific and Shared Fungal Assemblages

**Figure 8:**
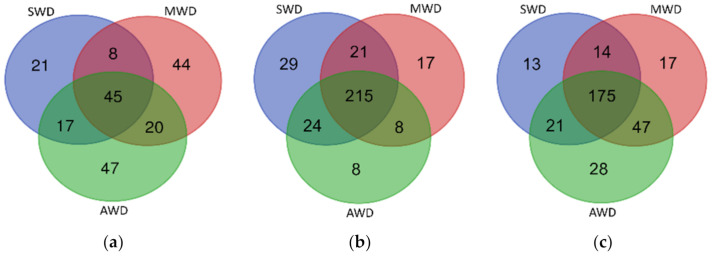
Venn diagram illustrating the overlap of the number of OTUs identified in the fungal microbiota among irrigation regimes (SWD: severe water deficit, MWD: moderate water deficit and AWD: absence of water deficit) in the (**a**) root, (**b**) rhizosphere, and (**c**) bulk soil.

The three soil-plant compartments showed specific fungal OTUs for each irrigation regimes and a cluster of shared OTUs. In the root, 22.3% of fungal OTUs were shared among irrigation regimes, while specific fungal OTUs associated with each irrigation regime ranged from 10.4% to 23.3% (Figure 8a). In the rhizosphere, specific fungal OTUs associated with irrigation regimes ranged from 2.5% to 9% whereas shared OTUs among irrigation regimes represented the 66.8% of the rhizosphere fungal communities (Figure 8b). In bulk soil, 55.6% of fungal OTUs were shared among irrigation regimes and specific OTUs associated with each irrigation regime ranged from 4.1% to 8.9% (Figure 8c). The OTUs that were unique in each of the irrigation regime within each soil-plant compartment are shown in Table S4.

### 3.7. High Level of Connectivity among Black-Foot Fungi in the Root

**Figure 9:**
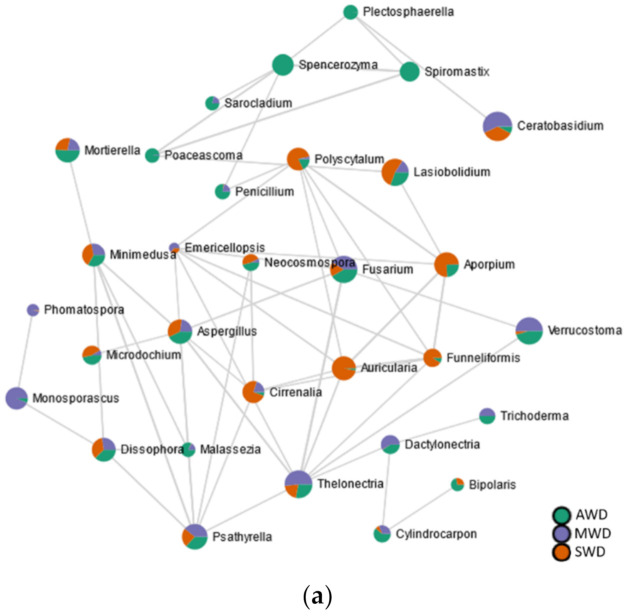

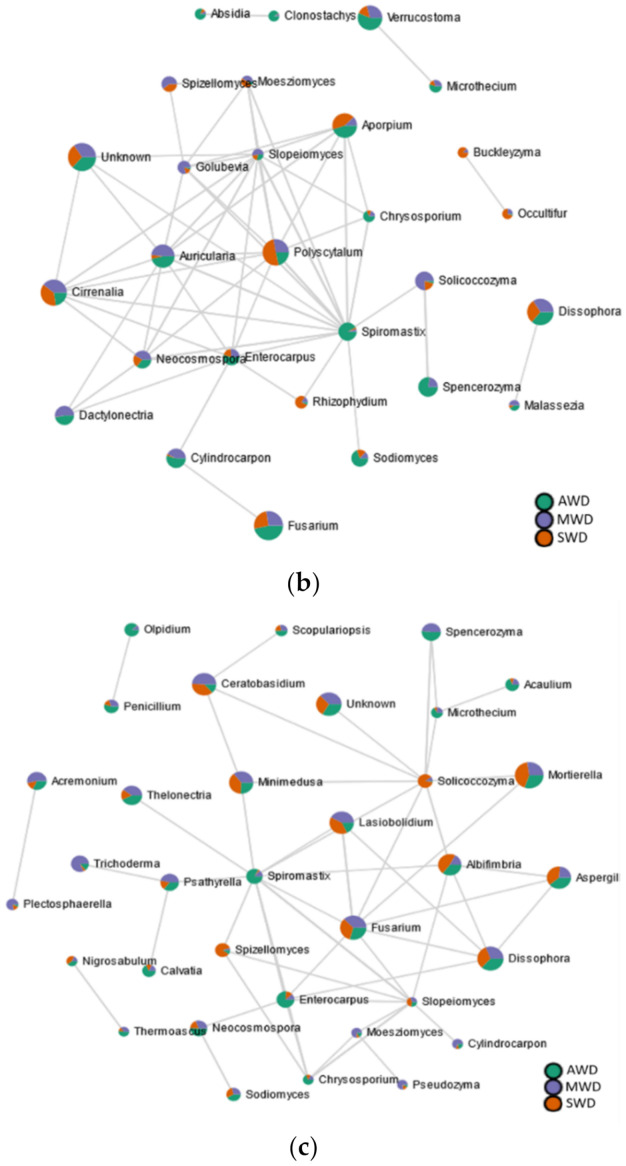
SparCC correlation analysis at genus level among irrigation regimes (SWD: severe water deficit, MWD: moderate water deficit, and AWD: absence of water deficit) in the (**a**) root, (**b**) rhizosphere, and (**c**) bulk soil.

A higher quantity of significant edges and connections was observed with rhizosphere (*n* = 116) compared to the root (*n* = 112) and the bulk soil (*n* = 102) (Figure 9; Table S5). In the root (Figure 9a), the black-foot fungal genera “Cylindrocarpon”, Dactylonectria, and Thelonectria correlated positively among them. The biocontrol agent Trichoderma correlated positively with Dactylonectria, while the AMF Funneliformis correlated negatively with Thelonectria. No correlations were established among black-foot fungi and/or biocontrol agents in both the rhizosphere (Figure 9b) or in the bulk soil (Figure 9c).

### 3.8. Water Deficit Affects Fungal Functionality in the Root

**Figure 10:**
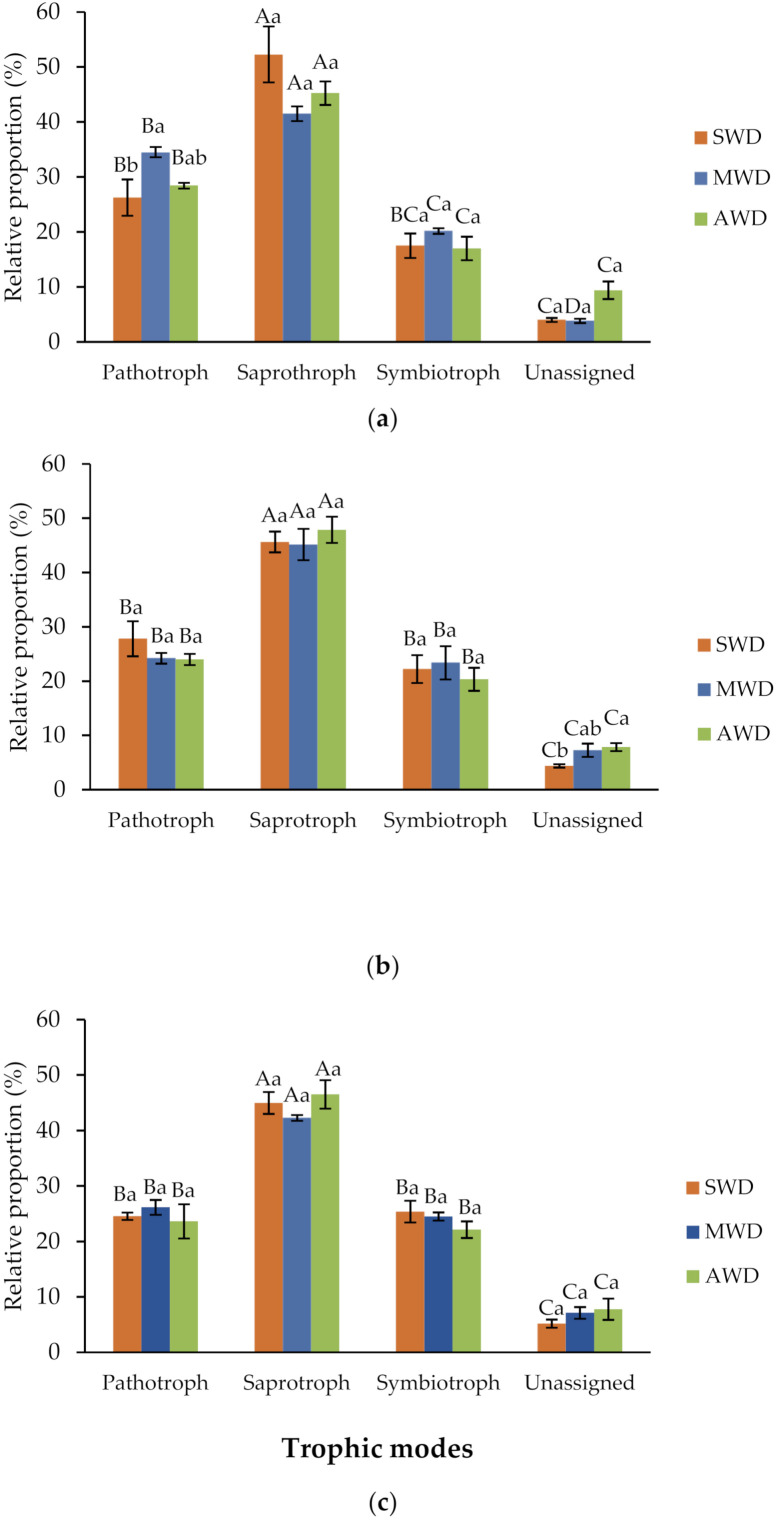
Variations in fungal function inferred by FUNGuild in in the (**a**) root, (**b**) rhizozphere and (**c**) bulk soil, at different irrigation regimes: severe water deficit (SWD), moderte water deficit (MWD), and absence of water deficit (AWD). Tukey’s test at *p*-value < 0.05 level. Means followed by the same letter do not differ significantly. Capital letters are for comparison of means among functional groups within each irrigation regime. Small letters are for comparison of means among irrigation regimes within each functional group.

Overall, the relative abundance of fungal OTUs identified as trophic modes with pathotrophs, saprotrophs, and symbiotrophs ranged from 90.6% to 96.2% in the root, 92.2% to 95.6% in the rhizosphere, and 92.2% to 94.8% in the bulk soil, while the remaining OTUs were unassigned (Figure 10). There were signficant differences in the relative proportion of fungal functions within each irrigation regimes in each soil-plant compartment (*p*-value < 0.05) (Table S6).

In the root, the trophic mode was dominated by saprothrophs, followed by pathotrophs and symbiotrophs, altough there were no significant differences between pathotrophs and symbiotrophs in the SWD treatment (*p*-value < 0.05). Pathotrophs were found at SWD treatment (26.3%) in a lower proportion compared with MWD (34.5%) (Figure 10a). In rhizosphere and bulk soil samples, the trophic mode was dominated by saprotrophs, followed by pathotrophs and symbiotrophs at all watering regimes, without significant differences between pathotrophs and symbiotrophs (*p*-value < 0.05) (Figure 10b,c).

Plant pathogens were the dominant taxa in the pathotroph group in the root and rhizosphere (Table S7). In the saprotroph group, undefined saptrotrophs were the dominant taxa in the three compartments at all irrigation regimes (Table S7). In the symbiotrophs group, endophytes were the most abundant taxa in the bulk soil and rhizosphere, whereas no differences were found between endophytes and arbuscular mycorrhiza at SWD and MWD regimes in the root (Table S7).

## 4. Discussion

This study focused on exploring the influence of different scenarios of soil water availability on the root endosphere, rhizosphere, and bulk soil fungal microbiome of grapevine, by ITS HTAS approach. We were particularly interested in understanding the impact of drought-stress on the root-associated fungal communities, with special attention to BFD fungal abundance.

The fungal community composition was influenced by the soil-plant compartment. Our study detected that fungal diversity decreased in the root compartment with respect to the the rhizosphere and bulk soil compartments, and this is in accordance with previous research aiming to decipher the bacterial and fungal microbiome of grapevine [26,27,28,30,57,58].

The major fungal phyla detected in our work were largely composed of Ascomycota and Basidiomycota, which accounted between 62 to 89% of the relative abundance in the soil-plant compartments, across the three watering scenarios. This taxonomic pattern is consistent with results obtained in previous studies that explored the belowground grapevine fungal microbiome, supporting the idea that the selective forces defining fungal root microbiome structure at a high taxonomic rank are constant under various environmental conditions [27,29,30,57,58,59,60,61,62].

The fungal phylum Mucoromycota was mostly found in the rhizosphere and bulk soil compared with the root endosphere, and was largely represented by the family Mortierellaceae, particularly by the genus *Mortierella*. Our results showed that *Mortierella* was one of the most abundant genera found in the rhizosphere and bulk soil, accounting for 9 to 17% and 14 to 20% of the relative abundance of all genera, respectively. Similar results were found in previous grapevine studies, in which Mortierellaceae was the most abundant family in the bulk soil and rhizosphere, and *Mortierella* was the most dominant genus in the root-zone soil and showed lower abundances in the plant-compartments (root, leaf, flower, and grape) [27,30,63]. The genus *Mortierella* is a phosphate solubilizing fungus which plays an important role in the phosphorus cycling in the rhizosphere [64]. Interestingly, the co-inoculation of *Mortierella* with an AMF showed a positive effect on enhancing plant growth and phosphorus uptake of avocado crop [65].

Our results indicated that the fungal microbiome diversity in root and bulk soil varied according to the sampling time, although this effect was not consistent in the rhizosphere. We detected an increase in fungal diversity towards the twelve-month sampling in root, whereas the opposite was observed in bulk soil. The 6-month sampling (June) coincides with the time after leaf senescence (late fall in southern hemisphere), whereas at the 12-month sampling (December), grapevines were in active vegetative development. Liu and Howell [30] observed that the grapevine associated microbiota is affected by the plant developmental stage throughout the growing season from flowering to harvest, in above- (grape and leaf) and belowground compartments, and suggested that veraison is the most distinct stage. In addition, they found that the fungal diversity fluctuation was similar in rhizosphere and root samples [30], reinforcing the idea that root microbiomes are partially derived from the rhizosphere and, in turn, that root and exudation and morphology profile can influence the composition of the rhizosphere microbiome [57,66]. On the other hand, in a previous research conducted in Spain, Berlanas et al. [29] observed a non-clear pattern of fluctuation of fungal diversity in the rhizosphere of vines grown in two vineyards of different geographical location, age, climate, and soil management practices. Year of sampling has also been pointed out as a major factor that can influence the diversity and composition of the microbiota in grapevine [29,63]. This phenomenon can be attributed to distinct root responses to different environmental factors, such as precipitation or temperature [29,67]. Further research is therefore needed to better understand shifts in fungal community composition throughout the annual growth cycle and how the year of sampling may influence the community succession.

The irrigation regimes strongly influenced fungal diversity and composition of the belowground compartments of grapevine. Overall, the major differences in fungal diversity were observed between the treatments of SWD and the full-watered condition (AWD). A decrease in the relative abundances of pathotrophs were predicted in roots at SWD. Although several previous studies have shown that drought influences the bacterial composition across many plant species [5,10,12,16,17,18,19,20,21], the overall impact of drought on the fungal grapevine microbiome had not yet been unravelled. Our results showed that diversity of OTUs significantly decreased towards the treatment of SWD in the three soil-plant compartments. A strong correlation between the water status (relative soil moisture and evaporation) and the grapevine fungal microbiome composition has been shown by Liu and Howell [30]. In contrast, Swift et al. [58] did not find a large impact of irrigation on patterns of grapevine microbial diversity, although a differential abundance of fungal and bacterial taxa varied as a consequence of the irrigation treatments. However, the amount of seasonal precipitation received during their experiment could have been enough to obscure some of the signal from the severe water stress [58]. Drought triggers a series of responses in plants, ranging from shifts in the root morphology to metabolic perturbations, which alter the root exudate profile and may also affect the belowground associated microbiome [5,12,30]. Under drought conditions, the plant root system is able to attract and favour the establishment of microorganisms, which may improve the ecosystem services required to support plant growth and development [68,69]. Interestingly, in SO_4_ rootstock, inoculation with plant growth promoting (PGP) bacteria contributed to enhance grapevine adaptation to drought through a water stress-induced promotion capacity, rather than a per se trait of the PGP bacteria tested [69].

Several genera contributed to the dissimilarities observed among the irrigation regimes in the three soil plant compartments, according to the Linear Discriminant Analysis Effect Size. For instance, a significant enrichment of the AMF genus *Funneliformis* was observed in root (at six-month sampling) and in rhizosphere samples at the condition of SWD. The genus *Funneliformis*, previously classified in the former genus *Glomus* sensu lato, is a fungus from the Glomeraceae family, the taxon that largely dominates the AMF communities detected in cultivated grapevine, and also in wild grapevine [62,70,71,72]. We also detected in our study the presence of the AMF *Rhizophagus* (Glomerales), *Acaulospora* (Diversisporales), and *Diversispora* (Diversisporales), previously reported in vineyards [73], but in very low abundances. The AMF-grapevine symbiosis provides several ecosystemic services for grapevine production, which may be of benefit in terms of adaptation to new challenges of pest management and climate change, such as increasing droughts [73]. AMF are an important groups of soil microorganisms which provide an increased interface between roots and soil, therefore improving grapevine growth and nutrition by enhancing soil nutrients uptake, as well as increasing tolerance to biotic and abiotic stresses, such as water stress [73]. Indeed, Donkó et al. [74] reported that the degree of grapevine mycorrhizal colonization was higher in drier soil areas in Hungary. Our results may suggest that grapevine mycorrhization is expected to naturally increase as a consequence of drought. Nevertheless, aspects concerning soil characteristics [70,75], vineyard agricultural practices, such as tillage, high fertilizers inputs [76], and pesticide application [77,78], as well as characteristics relating to the host, such as the rootstock genotype [79] and to a lesser extent the plant phenological stage [80], can greatly impact on AMF diversity and grapevine mycorrhization. Management practices that conserve the biodiversity of AMF in vineyards may be essential to profit from the ecosystem services concerning increased drought tolerance in the grapevine that AMF provides.

*Cylindrocarpon*-like asexual morphs associated with BFD, namely “*Cylindrocarpon*”, *Dactylonectria*, and *Thelonectria* showed significantly higher abundances at treatments of MWD and AWD of water deficit rather than at the treatment of SWD. Correlation network analysis also highlighted the high level of connectivity among black-foot fungi in the root under the same irrigation conditions. A wide diversity of *Cylindrocarpon*-like asexual morphs has been reported to co-exist on the same tissue [81], but their interactions have never been studied. Research on effects of co-infections on symptom expression among black-foot fungi and other grapevine trunk disease pathogens has been published. Grapevines infected with *Botryosphaeriaecea* spp. [82] or Petri disease pathogens [83] and black-foot fungi had increased disease incidence and severity than with single pathogen infections. In vitro assays showed that “*Cylindrocarpon*” spp. isolates showed reduced mycelial growth as water potential decreased in the culture medium [84]. It is widely recognized that BFD incidence is favored by poor drainage conditions and high moisture content of soil [34]. Our results suggest that extreme conditions of water deficit may be unfavorably for black-foot pathogens survival. Further long-term assays are necessary to evaluate if the lower presence of black-foot pathogens detected at the condition of SWD compared with the full-irrigated regime also implies a reduction in BFD severity and incidence in grapevine.

Another interesting hypothesis which might partially explain the lowest presence of black-foot pathogens observed in the root and rhizosphere at the SWD condition, could be the enrichment of AMF detected in this irrigation regime. The presence of AMF has been negatively correlated with pathotrophic fungi in wild grapevine [62]. Furthermore, some AMF are cataloged as biocontrol agents [85]. For instance, inoculation with *Rhizophagus irregularis* (syn. *Glomus intradices*), from Glomeraceae, reduced the disease severity and incidence of root lesions caused by black-foot pathogens on *Vitis* rupestris [86]. By contrast, the application of commercial AMF as a pre-planting strategy against black-foot fungi did not result in the suppression of disease incidence, but instead increased the abundance of the pathogens [87].

Several studies on biological control of BFD have evaluated the application of *Trichoderma* spp. in young vineyards and grapevine nurseries [88,89,90,91,92,93], but with inconsistent results. Overall, we detected the presence of the genus *Trichoderma* in a similar pattern of distribution that black-foot pathogens, with higher abundances at treatments of MWD and AWD, rather than at the treatment of SWD. Our results suggest that, in regions where drought events are expected to increase [1,2], the use of *Trichoderma*-based biological products against BFD, and other grapevine trunk disease fungi, will require further analysis to evaluate the success of *Trichoderma* spp. as biological control fungi in challenging environment conditions.

## 5. Conclusions

Our study demonstrated that water deficit influences fungal community dynamics of the belowground grapevine microbiome, with OTUs diversity significantly decreasing towards the treatment of SWD in the root endosphere, rhizosphere, and bulk soil. Black-foot fungi belonging to the genera “*Cylindrocarpon*”, *Dactylonectria*, and *Thelonectria* were severely affected by drought, and presented a high level of connectivity among them in the root under the same irrigation conditions. Other fungal genera, such as the AMF *Funneliformis* were enriched under extreme conditions (SWD), which would make these microorganisms viable, strong, and vital options for water stress mitigation in grapevine. Further studies focusing on examining the impact of SWD condition on belowground microbiomes of different grapevine rootstocks and rootstock/scion combinations, and in diverse soil types, will be indispensable to improve our understanding of how prolonged and more frequent drought events would affect the root-associated fungal microbiome on grapevine, and the consequences of altering the microbial terroir, including the abundance of potential soil-borne pathogens of grapevine.

## Figures and Tables

**Table 1:** Experimental factors predicting alpha diversity of bulk soil-, rhizosphere- and root-associated fungal communities.

Dataset	Factor	Indexes	
		Shannon	Chao1
Whole	Plant-soil compartments	*F* = 23.93 ***p* = 4.62 × 10^−^^10^**	*F* = 693.64 ***p* = 1.01 × 10^−91^**
Bulk soil	Sampling time	*F* = 4.93 ***p* = 7.69 × 10^−6^**	*F* = −1.43 *p* = 0.158
	Irrigation regime	*F* = 4.63 ***p* = 0.0130**	*F* = 8.58 ***p* = 0.0005**
	Sampling time × irrigation regime	*F* = 9.69 ***p* = 5.69 × 10^−7^**	*F* = 7.26 ***p* = 1.8 × 10^−5^**
Rhizosphere	Sampling time	*F* = 0.96 *p* = 0.3396	*F* = 0.22 *p* = 0.8267
	Irrigation regime	*F* = 12.73 ***p* = 2.22 × 10^−^^5^**	*F* = 10.15 ***p* = 0.0002**
	Sampling time × irrigation regime	*F* = 8.19 ***p* = 5.72 × 10^−^^6^**	*F* = 10.19 ***p* = 3.96 × 10^−7^**
Root	Sampling time	*F* = −4.16 ***p* = 0.0001**	*F* = −0.63 *p* = 0.5336
	Irrigation regime	*F* = 45.58 ***p* = 4.25 × 10^−^^13^**	*F* = 22.85 ***p* = 3.06 × 10^−8^**
	Sampling time × irrigation regime	*F* = 49.73 ***p* = 2.01 × 10^−2^^0^**	*F* = 8.93 ***p* = 1.99 × 10^−6^**
Bulk soil/Sampling time 1	Irrigation regime	*F* = 5.81 ***p* = 0.0069**	*F* = 3.36 ***p* = 0.0470**
Bulk soil/Sampling time 2	Irrigation regime	*F* = 4.37 ***p* = 0.0208**	*F* = 11.98 ***p* = 0.0001**
Root/Sampling time 1	Irrigation regime	*F* = 114.15 ***p* = 5.04 × 10^−^^15^**	*F* = 20.39 ***p* = 2.23 × 10^−^^6^**
Root/Sampling time 2	Irrigation regime	*F* = 116.18 ***p* = 3.96 × 10^−^^15^**	*F* = 26.81 ***p* = 1.74 × 10^−7^**

ANOVA, analysis of variance. Bold values indicate statistically significant results. *p*-value < 0.05. Sampling time 1: 6 months after the establishment of the irrigation regimes. Sampling time 2: 12 months after the establishment of the irri-gation regimes.

## Data Availability

Sequencing data are deposited under BioProject (https://www.ncbi.nlm.nih.gov/bioproject/) acc. No. PRJNA707008, where the SRA experiments are available by acc. Nos. SRX10263838–SRX10366007. The OTUs table, corresponding taxonomic classifications, and metadata for all samples used in this study were deposited in Zenodo (https://zenodo.org) acc. No. 5244774.

## Supplementary Materials

The following are available online at https://www.mdpi.com/article/10.3390/jof7090686/s1, Figure S1. Predawn leaf water potential at the irrigation regimes. Figure S2. Pruning weight at the irrigation regimes. Figure S3. Principal Coordinate Analysis (PCoA) based on Bray–Curtis dissimilarity metrics, showing the distance in the fungal communities among soil-plant compartments. Figure S4. Relative abundance of the most abundant (a) phyla, (b) families, and (c) genera in bulk-soil, rhizosphere, and root samples. Figure S5. Boxplot illustrating the differences in Shannon diversity measure of the fungal communities in (a) root and (b) bulk soil between 6- and 12-month sampling time. Figure S6. Principal Coordinate Analysis (PCoA) based on Bray–Curtis dissimilarity metrics, showing the distance in the fungal communities in (a) root, (b) rhizosphere, and (c) bulk soil between 6- and 12-month sampling time. Figure S7. Venn diagram illustrating the overlap of the OTUs identified in the fungal microbiota between 6- and 12-month sampling time in the (a) root, (b) rhizosphere, and (c) bulk-soil. Figure S8. Bar graph of LEfSe showing the most differentially taxa between 6- and 12-month sampling time in (a) root, (b) rhizosphere, and (c) bulk-soil. Figure S9. Relative abundance of the most abundant taxa in (a) root, (b) rhizosphere, and (c) bulk-soil in each watering regime. Figure S10. Bar graph of LEfSe showing the most differentially abundant taxa among the irrigation regimes in each sampling time in (a) root, (b) rhizosphere, and (c) bulk-soil. Table S1: Physicochemical properties of the grapevine nursery soil examined in this study. Table S2: Number of reads, total OTUs and alpha diversity indices. Table S3. Estimates of number of reads, sample coverage, and diversity indices at the genus level for fungal profiles. Table S4. Fungal OTUs that were unique in each irrigation regime in the soil-plant compartments. Table S5. SparCC correlation analysis at genus level in the bulk soil, rhizosphere, and root. Table S6. Relative proportion (%) of fungal function from soil-plant compartments at each irrigation regime inferred by FunGuild. Table S7. Compositions and relative abundance (%) of fungal functional groups (guild) inferred by FunGuild.
