# Supplementary material for: Drought Influences Fungal Community Dynamics in the Grapevine Rhizosphere and Root Microbiome
Source: J Fungi (Basel). 2021 Aug 25;7(9):686. doi: 10.3390/jof7090686 (PMC8468433; doi:10.3390/jof7090686)
Supplement: Supplementary file 1 [file jof-07-00686-s001.zip › jof-1334782-supplementary.pdf]

Table S1. Physicochemical properties of the grapevine nursery soil examined in this study.

	Grapevine nursery soil
Coordinates	34°34′48″ S, 56°17′50″ W
Location	Canelones
Altitude (m)	33
**Physicochemical properties**	
pH H_2O	6.0
K meq/100g	1.33
Mg meq/100g	3.3
Ca meq/100g	11.5
Na meq/100g	1.25
P Bray mg/Kg	137.0
N-NO3 mg/kg	52.0
SOM%	4.8
Clay%	28.9
Sand%	12.3
Silt%	58.8
Water content at field capacity %*	34.78

* % soil moisture by weight

Table S2. Number of reads, total OTUs and alpha diversity indices.

Index	Bulk soil					
	Sampling Time 1			Sampling Time 2		
	SWD[a]	MWD[b]	AWD[c]	SWD	MWD	AWD
Reads	23102.7 ± 416.8[d]	22924.8 ± 363.9	22429.8 ± 399.4	22859.1 ± 838.1	23285.8 ± 1057.6	28348.4 ± 722.4
OTUs	153	205	212	187	200	215
Chao1	67.06 ± 2.07	75.87 ± 2.98	72.1 ± 2.07	62.32 ± 2.41	70.17 ± 1.48	74.89 ± 1.45
Shannon	2.55 ± 0.03	2.84 ± 0.10	2.86 ± 0.08	2.41 ± 0.06	2.42 ± 0.03	2.57 ± 0.03
Index	Rhizosphere					
	Sampling Time 1			Sampling Time 2		
	SWD	MWD	AWD	SWD[e]	MWD[f]	AWD[g]
Reads	23863.8 ± 737.9	24057 ± 1222.0	23169.3 ± 531.0	24016.8 ± 2410.3	22719.8 ± 948.5	23993.4 ± 1055.0
OTUs	245	195	217	229	219	192
Chao1	87.37 ± 3.15	64.78 ± 1.28	77.14 ± 2.11	80.04 ± 2.63	78.79 ± 3.69	68.31 ± 2.25
Shannon	2.59 ± 0.04	2.44 ± 0.08	2.91 ± 0.08	2.45 ± 0.07	2.61 ± 0.05	2.70 ± 0.02
Index	Root					
	Sampling Time 1			Sampling Time 2		
	SWD	MWD	AWD[h]	SWD[i]	MWD	AWD[j]
Reads	23059.3 ± 391.9	22204.4 ± 309.6	22251.8 ± 371.7	22077.7 ± 629.8	26496.8 ± 685.3	30785.9 ± 845.2
OTUs	47	85	96	63	65	65
Chao1	17.88 ± 0.74	21.75 ± 1.26	28.40 ± 1.42	15.27 ± 0.45	21.17 ± 0.71	19.64 ± 0.56
Shannon	2.06 ± 0.03	2.11 ± 0.04	2.78 ± 0.04	2.13 ± 0.03	2.41 ± 0.02	2.61 ± 0.02

[a] Severe Water Deficit
[b] Moderate Water Deficit
[c] Absence of Water Deficit
[d] Values are the mean of 12 replicates
[e] Samples SU206 and SU223 were removed from the analysis due to the low number of sequences reads
[f] Sample SU245 was removed from the analysis due to the low number of sequences reads
[g] Samples SU289 and SU290 were removed from the analysis due to the low number of sequences reads
[h] Samples SU168 and SU174 were removed from the analysis due to the low number of sequences reads
[i] Sample SU214 was removed from the analysis due to the low number of sequences reads
[j] Sample SU283 was removed from the analysis due to the low number of sequences reads

Table S3. Estimates of number of reads, sample coverage and diversity indices at the genus level for fungal profiles.

Sample ID	Number of reads	Good's coverage (%)	Chao1 richness	Shannon diversity
SU3	24878	99.97	66.8±1.4	2.5
SU4	23362	99.99	53.0±0.0	2.4
SU5	20782	99.97	72.0±5.5	2.5
SU6	21329	99.99	74.0±2.3	2.6
SU7	22289	99.97	73.0±0.2	2.7
SU8	24341	99.98	66.5±2.6	2.4
SU11	23552	100.00	16.0±0.5	2.0
SU14	22100	99.99	15.0±0.0	1.9
SU16	22775	99.99	18.0±0.5	2.1
SU17	23200	99.99	15.0±0.0	1.9
SU18	21102	99.97	16.0±0.2	2.0
SU19	21094	99.98	17.0±0.2	1.9
SU23	22107	99.99	78.0±1.8	2.8
SU24	20820	99.96	86.3±5.5	2.5
SU25	22674	99.95	86.5±2.9	2.6
SU26	28490	99.96	95.2±7.4	2.2
SU27	21681	99.98	87.8±4.2	2.6
SU28	22186	99.98	80.9±1.4	2.6
SU33	24624	99.99	65.3±0.9	2.5
SU34	21563	99.99	54.0±0.0	2.7
SU35	23383	99.96	66.2±0.5	2.5
SU36	24500	99.99	73.0±0.2	2.5
SU37	24156	99.97	74.0±0.0	2.7
SU38	22025	99.97	67.0±1.8	2.6
SU43	24552	99.99	16.0±0.0	2.1
SU44	21713	99.99	18.0±0.5	2.1
SU45	23795	99.99	22.0±0.2	2.1
SU46	24193	100.00	22.5±2.6	2.2
SU47	25385	99.99	20.0±0.5	2.2
SU48	23250	99.99	19.0±0.5	2.1
SU53	23306	99.98	78.0±0.0	2.6
SU54	22080	99.96	112.0±10.0	2.7
SU55	24762	99.97	88.3±0.7	2.6
SU56	28583	99.96	100.4±5.5	2.7
SU57	24267	99.95	72.1±5.0	2.5
SU58	25410	99.94	83.1±4.0	2.5
SU63	24236	99.97	67.3±0.9	2.9
SU64	23698	99.99	79.2±1.8	3.1
SU65	21659	99.99	77.0±0.0	3.1
SU66	22208	99.98	92.8±4.2	2.9
SU67	20778	99.94	76.1±2.5	2.8
SU68	23147	99.99	67.6±1.2	2.9
SU73	22580	99.97	28.0±1.8	2.1
SU74	21579	99.99	24.5±2.6	2.3
SU75	22201	99.99	18.5±1.3	1.9

SU76	21264	99.98	16.0±0.0	2.0
SU77	20731	99.99	19.0±0.5	2.1
SU78	22232	100.00	17.0±0.2	2.1
SU83	24288	99.99	62.3±0.9	2.6
SU84	22429	99.97	67.9±2.3	2.7
SU85	21801	99.99	55.0±0.1	2.5
SU86	23695	99.97	62.0±3.2	2.6
SU87	30673	99.96	66.4±0.8	2.4
SU88	22445	99.99	64.0±2.9	2.6
SU98	25128	99.96	82.7±2.0	3.3
SU99	22284	100.00	77.3±0.7	3.1
SU100	22103	99.97	91.2±0.6	2.9
SU101	22773	99.97	76.8±2.8	2.7
SU102	24454	99.99	64.5±2.6	2.5
SU103	22629	99.98	58.0±0.0	2.0
SU107	21738	99.99	28.0±1.8	2.1
SU108	22219	99.99	28.0±4.6	2.4
SU109	20824	99.98	21.0±1.8	2.0
SU110	23451	99.99	22.0±0.2	2.2
SU111	23515	99.99	20.0±0.5	2.1
SU112	24119	99.99	19.0±0.0	2.1
SU118	21202	100.00	62.8±1.4	2.3
SU119	21186	99.99	65.0±4.2	2.6
SU120	20658	99.99	64.0±0.2	2.5
SU121	22743	99.99	65.1±0.5	2.5
SU122	34634	99.98	71.3±4.1	1.7
SU123	22930	99.96	71.5±2.9	2.1
SU126	21900	99.99	73.0±13.2	2.5
SU130	21708	99.98	62.0±0.0	2.9
SU131	22637	99.99	65.0±3.4	2.7
SU132	22867	99.99	73.0±0.1	2.9
SU133	22318	99.94	76.3±1.7	2.9
SU134	23056	99.99	75.2±1.8	2.9
SU135	21977	99.97	69.5±4.1	2.5
SU136	23981	100.00	25.0±0.0	2.6
SU138	21227	100.00	26.0±0.0	2.8
SU139	22792	99.99	28.0±2.3	2.7
SU140	21706	100.00	28.0±0.0	2.8
SU141	21726	100.00	24.0±0.0	2.8
SU142	24609	100.00	21.0±0.0	2.8
SU147	24581	99.96	70.8±2.0	2.4
SU148	23010	99.97	82.1±4.7	2.7
SU150	27500	99.94	73.2±2.3	3.1
SU151	24965	99.94	84.8±5.8	2.5
SU152	21854	99.99	71.1±0.5	3.0
SU153	22850	99.97	85.0±10.8	3.1
SU157	23062	99.99	69.0±0.0	3.3
SU158	26107	99.99	68.0±0.0	3.0
SU159	20574	99.98	74.5±3.2	2.5

SU160	21810	99.99	69.2±0.6	3.1
SU161	21142	99.97	90.5±2.2	3.1
SU166	21744	100.00	31.0±0.5	2.9
SU169	21585	100.00	34.0±0.0	2.9
SU172	21154	100.00	34.0±2.3	2.6
SU175	21994	99.99	33.0±0.0	3.0
SU177	22028	99.94	79.8±2.8	2.9
SU179	20904	99.97	88.0±2.6	3.1
SU180	23800	99.99	63.0±0.5	2.9
SU182	22331	99.97	79.5±6.4	2.8
SU183	23680	99.97	75.3±0.9	3.1
SU184	20529	100.00	73.0±0.2	3.3
SU186	21970	99.98	49.8±1.4	2.5
SU187	22833	99.94	66.5±1.9	2.4
SU188	22289	99.97	57.2±4.3	2.4
SU189	23412	99.96	69.0±8.0	2.4
SU190	21706	99.96	51.8±1.4	2.4
SU192	31736	99.96	53.7±2.0	1.9
SU193	20213	100.00	13.0±0.0	2.0
SU194	20565	99.99	16.0±0.0	2.2
SU195	20195	100.00	17.0±0.0	2.0
SU196	23293	99.99	15.0±2.3	2.2
SU198	21022	100.00	14.0±0.0	2.0
SU199	20002	99.99	13.0±0.0	2.1
SU200	25087	99.92	90.7±3.8	2.8
SU201	25492	99.95	66.1±2.3	2.6
SU202	15736	99.94	84.5±4.1	2.6
SU203	23892	99.94	77.5±4.5	2.7
SU204	15869	99.97	74.5±1.9	2.4
SU207	22915	99.95	74.0±2.6	2.7
SU208	21617	99.94	74.9±2.3	2.6
SU209	22387	99.96	65.0±1.6	2.3
SU210	20762	99.96	61.0±1.8	2.6
SU211	21779	99.94	58.6±2.8	2.4
SU212	20903	99.95	66.4±1.9	2.4
SU215	25791	99.99	17.0±0.0	2.1
SU216	25747	99.99	16.0±0.0	2.1
SU217	22074	100.00	15.0±0.0	2.1
SU218	22251	99.98	15.0±0.2	2.2
SU219	21702	99.98	17.0±0.0	2.3
SU222	42442	99.90	73.1±3.4	2.1
SU224	20636	99.96	72.6±2.5	2.0
SU225	21343	99.96	86.5±4.1	2.5
SU226	28382	99.96	86.4±5.5	2.6
SU227	21289	99.94	88.5±4.5	2.4
SU229	26180	99.96	68.5±2.2	2.5
SU230	25129	99.99	61.0±0.2	2.6
SU231	33461	99.90	74.0±13.2	2.4
SU232	21980	99.97	72.7±2.2	2.6

SU233	22855	99.94	66.7±2.2	2.4
SU234	20907	99.96	80.6±5.3	2.6
SU236	26697	99.99	21.0±0.2	2.5
SU237	23264	99.98	22.0±0.5	2.4
SU238	28513	99.99	22.0±0.0	2.3
SU239	26429	99.99	24.0±0.0	2.5
SU240	21113	99.99	26.0±0.5	2.6
SU241	26670	99.99	23.0±0.5	2.4
SU243	22142	99.95	74.2±7.4	2.5
SU244	21991	99.94	77.5±2.6	2.7
SU246	24709	99.96	73.4±5.5	2.6
SU247	24709	99.94	73.4±5.5	2.6
SU248	22029	99.95	65.0±6.6	2.6
SU250	22955	99.97	72.8±1.3	2.3
SU251	20544	99.95	66.3±5.4	2.3
SU252	20209	99.95	73.5±1.3	2.4
SU253	22302	99.94	72.1±0.4	2.3
SU254	21452	99.97	68.3±0.7	2.4
SU255	21455	99.98	65.8±1.4	2.3
SU257	27601	99.99	17.0±0.2	2.4
SU258	28745	99.98	20.0±2.3	2.3
SU259	28713	99.99	21.0±0.5	2.3
SU260	28908	99.98	19.0±0.0	2.5
SU261	25658	99.99	20.0±0.0	2.4
SU262	25650	99.99	19.0±0.0	2.4
SU264	27765	99.97	94.5±10.6	3.0
SU265	17036	99.94	80.3±2.6	2.7
SU266	21352	99.97	73.4±7.3	2.4
SU267	18387	99.97	95.4±12.6	2.6
SU268	23950	99.93	98.0±11.0	2.7
SU269	25848	99.94	61.4±5.5	2.6
SU271	31009	99.96	78.5±3.7	2.7
SU272	27626	99.92	73.1±4.7	2.7
SU273	30232	99.91	71.2±1.5	2.6
SU274	29494	99,94	81.0±8.9	2.5
SU275	29105	99.96	82.0±8.0	2.5
SU276	25957	99.95	77.0±8.5	2.4
SU278	30265	99.99	19.0±0.5	2.6
SU279	28055	100.00	17.0±0.0	2.6
SU280	32832	100.00	19.0±0.5	2.7
SU281	29860	100.00	17.0±0.0	2.5
SU282	33962	100.00	18.0±0.0	2.7
SU285	25278	99.96	80.5±6.4	2.8
SU286	15806	99.94	62.9±2.3	2.7
SU287	25581	99.96	65.4±5.5	2.7
SU288	28364	99.92	67.1±5.0	2.7
SU292	33713	99.96	71.2±4.3	2.4
SU293	26410	99.96	69.4±0.8	2.7
SU294	27041	99.94	69.7±2.2	2.6

SU295	26558	99.94	76.3±0.7	2.6
SU296	24790	99.94	68.2±0.5	2.6
SU298	28246	99.92	81.1±7.2	2.6
SU299	30218	100.00	19.0±0.0	2.6
SU300	27367	99.99	21.0±0.0	2.6
SU301	29303	99.99	22.0±0.5	2.6
SU302	27560	100.00	21.0±0.0	2.7
SU303	33656	100.00	22.0±0.0	2.7
SU304	35567	100.00	21.0±0.0	2.6
SU307	26928	99.96	68.5±6.6	2.6
SU308	23409	99.94	64.1±4.6	2.7
SU309	24066	99.97	82.2±7.4	2.8
SU310	23826	99.94	62.4±1.9	2.7
SU311	23429	99.96	65.9±2.3	2.7
SU312	23247	99.96	64.0±3.4	2.7

Table S4. Fungal OTUs that were unique in each irrigation regime in the soil-plant compartments.

Bulk soil			Rhizosphere			Root		
SWD[a]	MWD[b]	AWD[c]	SWD	MWD	AWD	SWD	MWD	AWD
Alfaria	*Achaetomium*	*Acrophialophora*	*Bacillicladium*	*Achroiostachys*	*Allomyces*	*Achroiostachys*	*Absidia*	*Campylocarpon*
Allomyces	*Achroiostachys*	*Alnicola*	*Bolbitius*	*Alfaria*	*Coniolariella*	*Allomyces*	*Acaulospora*	*Conocybe*
Arxiella	*Bolbitius*	*Bacillicladium*	*Burgoa*	*Auxarthronopsis*	*Echinusitheca*	*Ascotricha*	*Achaetomium*	*Cystobasidium*
Auxarthronopsis	*Echinusitheca*	*Candida*	*Chordomyces*	*Flavodon*	*Geminibasidium*	*Bolbitius*	*Alfaria*	*Gymnoascus*
Holtermanniella	*Fellomyces*	*Colletotrichum*	*Cladorrhinum*	*Fumagopsis*	*Hydnangium*	*Bullera*	*Alnicola*	*Heteroradulum*
Hydnangium	*Kurtzmaniella*	*Coprinus*	*Cyberlindnera*	*Fusicolla*	*Parawiesneriomyces*	*Byssochlamys*	*Anthracocystis*	*Holtermanniella*
Myriococcum	*Laetisaria*	*Coralloidiomyces*	*Diversispora*	*Hormodochis*	*Pezicula*	*Calvatia*	*Arachnotheca*	*Hyaloseta*
Paecilomyces	*Lectera*	*Diatrypella*	*Entyloma*	*Lobulomyces*	*Sistotrema*	*Chlorophyllum*	*Arizonaphlyctis*	*Ijuhya*
Pezicula	*Mucidula*	*Drechmeria*	*Harposporium*	*Marasmius*		*Gymnascella*	*Arthrocladium*	*Laetisaria*
Phaeoannellomyces	*Phialocephala*	*Elsinoe*	*Holtermanniella*	*Melanoleuca*		*Hapsidospora*	*Atractiella*	*Leucoagaricus*
Phaeophlebiopsis	*Pseudophialophora*	*Hyphodontia*	*Kurtzmaniella*	*Mucidula*		*Hyalorbilia*	*Auxarthronopsis*	*Lindtneria*
Pluteus	*Remispora*	*Kalmanozyma*	*Laccaria*	*Phaeoannellomyces*		*Kraurogymnocarpa*	*Buckleyzyma*	*Lipomyces*
Tulasnella	*Rhizophagus*	*Laccaria*	*Mycena*	*Phialocephala*		*Lentithecium*	*Classicula*	*Lobulomyces*
	Sistotrema	*Lentithecium*	*Myriococcum*	*Pleiocarpon*		*Metapochonia*	*Climacocystis*	*Marasmius*
	Sporothrix	*Marasmius*	*Phylliscum*	*Remispora*		*Minimelanolocus*	*Coprinellus*	*Matsushimaea*
	Sterigmatomyces	*Megasporoporia*	*Pluteus*	*Saccharomyces*		*Neoidriella*	*Coralloidiomyces*	*Megasporoporia*
	Trametes	*Mycena*	*Pseudophialophora*	*Thyronectria*		*Olpidium*	*Currahmyces*	*Meira*
		Mycoleptodiscus	*Pseudothielavia*			*Phaeoacremonium*	*Cyberlindnera*	*Microascus*
		Neopaxillus	*Ramariopsis*			*Phylliscum*	*Debaryomyces*	*Microthecium*
		Parawiesneriomyces	*Rhinocladiella*			*Spizellomyces*	*Dimorphiseta*	*Mucor*
		Pleohelicoon	*Rosasphaeria*			*Synfenestella*	*Eichleriella*	*Papiliotrema*
		Polytolypa	*Sporidiobolus*				*Elsinoe*	*Phaeophlebiopsis*
		Ramariopsis	*Stagonospora*				*Epichloe*	*Pluteus*
		Saccharomyces	*Subulicystidium*				*Erythrobasidium*	*Pochonia*
		Sporisorium	*Synfenestella*				*Farysia*	*Polycephalomyces*
		Taphrina	*Trichosporonaceae*				*Geastrum*	*Porpoloma*
		Tilletiopsis	*Tulasnella*				*Gilbertella*	*Pseudallescheria*
		Wojnowiciella	*Tygervalleyomyces*				*Golubevia*	*Pseudohyphozyma*
			Vaginatispora				*Harposporium*	*Pseudozyma*
							Lindtneria	*Psilocybe*
							Meyerozyma	*Rasamsonia*
							Microstroma	*Resupinatus*

Naganishia	*Rhizophagus*
Neostrelitziana	*Sampaiozyma*
Paecilomyces	*Scedosporium*
Peniophora	*Sistotremastrum*
Phialocephala	*Spiromastix*
Remispora	*Sporobolomyces*
Sporothrix	*Sterigmatomyces*
Tetracoccosporium	*Striatibotrys*
Tulasnella	*Talaromyces*
Tulostoma	*Trematosphaeria*
Udeniozyma	*Trichomerium*
Vaginatispora	*Trichosporonaceae*
	Veronaea
	Wallemia
	Wojnowiciella

[a] Severe Water Deficit
[b] Moderate Water Deficit
[c] Absence of Water Deficit

Table S5. SparCC correlation analysis at genus level in the bulk soil, rhizosphere and root.

Bulk soil				Rhizosphere				Root			
Taxon1	Taxon2	Correlation	*p*-value	Taxon1	Taxon2	Correlation	*p*-value	Taxon1	Taxon2	Correlation	*p*-value
Acaulium	*Microthecium*	0.5704	0.0099	*Absidia*	*Clonostachys*	0.5493	0.0099	*Aporpium*	*Auricularia*	0.8039	0.0099
Acremonium	*Plectosphaerella*	0.5569	0.0099	*Aporpium*	*Auricularia*	0.6864	0.0099	*Aporpium*	*Emericellopsis*	0.5153	0.0099
Albifimbria	*Aspergillus*	0.6357	0.0099	*Aporpium*	*Chrysosporium*	-0.5182	0.0099	*Aporpium*	*Funneliformis*	0.7903	0.0099
Albifimbria	*Dissophora*	0.5247	0.0099	*Aporpium*	*Golubevia*	-0.5347	0.0099	*Aporpium*	*Lasiobolidium*	0.5147	0.0099
Albifimbria	*Slopeiomyces*	0.5078	0.0099	*Aporpium*	*Polyscytalum*	0.5732	0.0099	*Aporpium*	*Polyscytalum*	0.695	0.0099
Albifimbria	*Solicoccozyma*	0.6486	0.0099	*Aporpium*	*Slopeiomyces*	0.5956	0.0099	*Aspergillus*	*Cirrenalia*	-0.5248	0.0099
Albifimbria	*Spiromastix*	-0.5157	0.0099	*Aporpium*	*Spiromastix*	-0.7376	0.0099	*Aspergillus*	*Emericellopsis*	-0.5029	0.0099
Aspergillus	*Albifimbria*	0.6357	0.0099	*Auricularia*	*Aporpium*	0.6864	0.0099	*Aspergillus*	*Fusarium*	0.6541	0.0099
Aspergillus	*Dissophora*	0.531	0.0099	*Auricularia*	*Cirrenalia*	0.549	0.0099	*Aspergillus*	*Malassezia*	-0.5123	0.0099
Aspergillus	*Fusarium*	0.5694	0.0099	*Auricularia*	*Dactylonectria*	0.5232	0.0099	*Aspergillus*	*Microdochium*	-0.5006	0.0099
Calvatia	*Psathyrella*	0.5562	0.0099	*Auricularia*	*Enterocarpus*	0.55	0.0099	*Aspergillus*	*Minimedusa*	0.6593	0.0099
Ceratobasidium	*Minimedusa*	0.5631	0.0099	*Auricularia*	*Golubevia*	-0.5924	0.0099	*Aspergillus*	*Psathyrella*	0.6552	0.0099
Ceratobasidium	*Scopulariopsis*	0.5053	0.0099	*Auricularia*	*Neocosmospora*	0.5914	0.0099	*Aspergillus*	*Thelonectria*	0.7568	0.0099
Ceratobasidium	*Solicoccozyma*	0.5834	0.0099	*Auricularia*	*Polyscytalum*	0.6131	0.0099	*Auricularia*	*Aporpium*	0.8039	0.0099
Chrysosporium	*Enterocarpus*	-0.5663	0.0099	*Auricularia*	*Slopeiomyces*	0.6751	0.0099	*Auricularia*	*Cirrenalia*	0.5246	0.0099
Chrysosporium	*Moesziomyces*	0.5425	0.0099	*Auricularia*	*Spiromastix*	-0.7853	0.0099	*Auricularia*	*Emericellopsis*	0.6216	0.0099
Chrysosporium	*Slopeiomyces*	-0.7904	0.0099	*Auricularia*	*Unknown*	0.557	0.0099	*Auricularia*	*Funneliformis*	0.8496	0.0099
Chrysosporium	*Spiromastix*	0.7599	0.0099	*Buckleyzyma*	*Occultifur*	0.5155	0.0099	*Auricularia*	*Polyscytalum*	0.5524	0.0099
Chrysosporium	*Spizellomyces*	0.6206	0.0099	*Chrysosporium*	*Aporpium*	-0.5182	0.0099	*Auricularia*	*Thelonectria*	-0.6497	0.0099
Cylindrocarpon	*Slopeiomyces*	-0.5264	0.0099	*Chrysosporium*	*Polyscytalum*	-0.5673	0.0099	*Bipolaris*	*Cylindrocarpon*	-0.5001	0.0099
Dissophora	*Albifimbria*	0.5247	0.0099	*Chrysosporium*	*Slopeiomyces*	-0.5718	0.0099	*Ceratobasidium*	*Plectosphaerella*	-0.5808	0.0099
Dissophora	*Aspergillus*	0.531	0.0099	*Chrysosporium*	*Spiromastix*	0.6547	0.0099	*Cirrenalia*	*Aspergillus*	-0.5248	0.0099
Dissophora	*Enterocarpus*	0.5749	0.0099	*Cirrenalia*	*Auricularia*	0.549	0.0099	*Cirrenalia*	*Auricularia*	0.5246	0.0099
Dissophora	*Fusarium*	0.6267	0.0099	*Cirrenalia*	*Enterocarpus*	0.6174	0.0099	*Cirrenalia*	*Funneliformis*	0.5339	0.0099
Dissophora	*Lasiobolidium*	0.5665	0.0099	*Cirrenalia*	*Neocosmospora*	0.6008	0.0099	*Cirrenalia*	*Neocosmospora*	-0.6248	0.0099
Enterocarpus	*Chrysosporium*	-0.5663	0.0099	*Cirrenalia*	*Polyscytalum*	0.5706	0.0099	*Cirrenalia*	*Psathyrella*	-0.5547	0.0099
Enterocarpus	*Dissophora*	0.5749	0.0099	*Cirrenalia*	*Slopeiomyces*	0.648	0.0099	*Cylindrocarpon*	*Bipolaris*	-0.5001	0.0099
Enterocarpus	*Fusarium*	0.5324	0.0099	*Cirrenalia*	*Spiromastix*	-0.6191	0.0099	*Cylindrocarpon*	*Dactylonectria*	0.529	0.0099
Enterocarpus	*Neocosmospora*	0.6053	0.0099	*Cirrenalia*	*Unknown*	0.5029	0.0099	*Dactylonectria*	*Cylindrocarpon*	0.529	0.0099
Enterocarpus	*Slopeiomyces*	0.6792	0.0099	*Clonostachys*	*Absidia*	0.5493	0.0099	*Dactylonectria*	*Thelonectria*	0.5102	0.0099
Enterocarpus	*Spiromastix*	-0.6172	0.0099	*Cylindrocarpon*	*Enterocarpus*	0.5825	0.0099	*Dactylonectria*	*Trichoderma*	0.511	0.0099
Fusarium	*Aspergillus*	0.5694	0.0099	*Cylindrocarpon*	*Fusarium*	0.5216	0.0099	*Dissophora*	*Malassezia*	-0.6225	0.0099

Fusarium	*Dissophora*	0.6267	0.0099	*Dactylonectria*	*Auricularia*	0.5232	0.0099	*Dissophora*	*Minimedusa*	0.6389	0.0099
Fusarium	*Enterocarpus*	0.5324	0.0099	*Dactylonectria*	*Enterocarpus*	0.5245	0.0099	*Dissophora*	*Monosporascus*	-0.5595	0.0099
Fusarium	*Lasiobolidium*	0.7815	0.0099	*Dactylonectria*	*Neocosmospora*	0.6283	0.0099	*Dissophora*	*Psathyrella*	0.6333	0.0099
Fusarium	*Mortierella*	0.5663	0.0099	*Dissophora*	*Malassezia*	0.5006	0.0099	*Emericellopsis*	*Aporpium*	0.5153	0.0099
Fusarium	*Slopeiomyces*	0.5507	0.0099	*Enterocarpus*	*Auricularia*	0.55	0.0099	*Emericellopsis*	*Aspergillus*	-0.5029	0.0099
Fusarium	*Solicoccozyma*	0.5769	0.0099	*Enterocarpus*	*Cirrenalia*	0.6174	0.0099	*Emericellopsis*	*Auricularia*	0.6216	0.0099
Fusarium	*Spiromastix*	-0.5423	0.0099	*Enterocarpus*	*Cylindrocarpon*	0.5825	0.0099	*Emericellopsis*	*Funneliformis*	0.6342	0.0099
Lasiobolidium	*Dissophora*	0.5665	0.0099	*Enterocarpus*	*Dactylonectria*	0.5245	0.0099	*Emericellopsis*	*Fusarium*	-0.5976	0.0099
Lasiobolidium	*Fusarium*	0.7815	0.0099	*Enterocarpus*	*Neocosmospora*	0.6078	0.0099	*Emericellopsis*	*Polyscytalum*	0.5661	0.0099
Lasiobolidium	*Spiromastix*	-0.5251	0.0099	*Enterocarpus*	*Polyscytalum*	0.5502	0.0099	*Emericellopsis*	*Thelonectria*	-0.5674	0.0099
Microthecium	*Acaulium*	0.5704	0.0099	*Enterocarpus*	*Rhizophydium*	-0.5521	0.0099	*Funneliformis*	*Aporpium*	0.7903	0.0099
Microthecium	*Solicoccozyma*	-0.5104	0.0099	*Enterocarpus*	*Slopeiomyces*	0.6156	0.0099	*Funneliformis*	*Auricularia*	0.8496	0.0099
Microthecium	*Spencerozyma*	0.5266	0.0099	*Enterocarpus*	*Spiromastix*	-0.5635	0.0099	*Funneliformis*	*Cirrenalia*	0.5339	0.0099
Minimedusa	*Ceratobasidium*	0.5631	0.0099	*Fusarium*	*Cylindrocarpon*	0.5216	0.0099	*Funneliformis*	*Emericellopsis*	0.6342	0.0099
Minimedusa	*Solicoccozyma*	0.6639	0.0099	*Golubevia*	*Aporpium*	-0.5347	0.0099	*Funneliformis*	*Polyscytalum*	0.5772	0.0099
Minimedusa	*Spiromastix*	-0.5969	0.0099	*Golubevia*	*Auricularia*	-0.5924	0.0099	*Funneliformis*	*Thelonectria*	-0.539	0.0099
Moesziomyces	*Chrysosporium*	0.5425	0.0099	*Golubevia*	*Moesziomyces*	0.5495	0.0099	*Fusarium*	*Aspergillus*	0.6541	0.0099
Moesziomyces	*Pseudozyma*	0.5644	0.0099	*Golubevia*	*Polyscytalum*	-0.5758	0.0099	*Fusarium*	*Emericellopsis*	-0.5976	0.0099
Moesziomyces	*Slopeiomyces*	-0.5899	0.0099	*Golubevia*	*Slopeiomyces*	-0.5766	0.0099	*Fusarium*	*Polyscytalum*	-0.533	0.0099
Mortierella	*Fusarium*	0.5663	0.0099	*Golubevia*	*Spiromastix*	0.6808	0.0099	*Fusarium*	*Thelonectria*	0.912	0.0099
Mortierella	*Solicoccozyma*	0.5315	0.0099	*Golubevia*	*Spizellomyces*	0.5278	0.0099	*Fusarium*	*Verrucostoma*	0.5282	0.0099
Neocosmospora	*Enterocarpus*	0.6053	0.0099	*Malassezia*	*Dissophora*	0.5006	0.0099	*Lasiobolidium*	*Aporpium*	0.5147	0.0099
Neocosmospora	*Sodiomyces*	0.6406	0.0099	*Microthecium*	*Verrucostoma*	0.569	0.0099	*Lasiobolidium*	*Poaceascoma*	-0.5526	0.0099
Nigrosabulum	*Thermoascus*	0.6	0.0099	*Moesziomyces*	*Golubevia*	0.5495	0.0099	*Malassezia*	*Aspergillus*	-0.5123	0.0099
Olpidium	*Penicillium*	0.5207	0.0099	*Moesziomyces*	*Polyscytalum*	-0.5165	0.0099	*Malassezia*	*Dissophora*	-0.6225	0.0099
Penicillium	*Olpidium*	0.5207	0.0099	*Moesziomyces*	*Slopeiomyces*	-0.5564	0.0099	*Malassezia*	*Minimedusa*	-0.6099	0.0099
Plectosphaerella	*Acremonium*	0.5569	0.0099	*Moesziomyces*	*Spiromastix*	0.6601	0.0099	*Malassezia*	*Psathyrella*	-0.5497	0.0099
Psathyrella	*Calvatia*	0.5562	0.0099	*Moesziomyces*	*Spizellomyces*	0.5191	0.0099	*Microdochium*	*Aspergillus*	-0.5006	0.0099
Psathyrella	*Spiromastix*	0.5421	0.0099	*Neocosmospora*	*Auricularia*	0.5914	0.0099	*Minimedusa*	*Aspergillus*	0.6593	0.0099
Psathyrella	*Trichoderma*	0.5924	0.0099	*Neocosmospora*	*Cirrenalia*	0.6008	0.0099	*Minimedusa*	*Dissophora*	0.6389	0.0099
Pseudozyma	*Moesziomyces*	0.5644	0.0099	*Neocosmospora*	*Dactylonectria*	0.6283	0.0099	*Minimedusa*	*Malassezia*	-0.6099	0.0099
Scopulariopsis	*Ceratobasidium*	0.5053	0.0099	*Neocosmospora*	*Enterocarpus*	0.6078	0.0099	*Minimedusa*	*Mortierella*	0.5438	0.0099
Slopeiomyces	*Albifimbria*	0.5078	0.0099	*Neocosmospora*	*Polyscytalum*	0.6054	0.0099	*Minimedusa*	*Psathyrella*	0.7953	0.0099
Slopeiomyces	*Chrysosporium*	-0.7904	0.0099	*Neocosmospora*	*Slopeiomyces*	0.7164	0.0099	*Monosporascus*	*Dissophora*	-0.5595	0.0099
Slopeiomyces	*Cylindrocarpon*	-0.5264	0.0099	*Neocosmospora*	*Spiromastix*	-0.7184	0.0099	*Monosporascus*	*Phomatospora*	0.5714	0.0099
Slopeiomyces	*Enterocarpus*	0.6792	0.0099	*Occultifur*	*Buckleyzyma*	0.5155	0.0099	*Mortierella*	*Minimedusa*	0.5438	0.0099

Slopeiomyces	*Fusarium*	0.5507	0.0099
Slopeiomyces	*Moesziomyces*	-0.5899	0.0099
Slopeiomyces	*Spiromastix*	-0.8402	0.0099
Slopeiomyces	*Spizellomyces*	-0.6129	0.0099
Sodiomyces	*Neocosmospora*	0.6406	0.0099
Solicoccozyma	*Albifimbria*	0.6486	0.0099
Solicoccozyma	*Ceratobasidium*	0.5834	0.0099
Solicoccozyma	*Fusarium*	0.5769	0.0099
Solicoccozyma	*Microthecium*	-0.5104	0.0099
Solicoccozyma	*Minimedusa*	0.6639	0.0099
Solicoccozyma	*Mortierella*	0.5315	0.0099
Solicoccozyma	*Spencerozyma*	-0.7287	0.0099
Solicoccozyma	*Spiromastix*	-0.5835	0.0099
Solicoccozyma	*Unknown*	0.5399	0.0099
Spencerozyma	*Microthecium*	0.5266	0.0099
Spencerozyma	*Solicoccozyma*	-0.7287	0.0099
Spiromastix	*Albifimbria*	-0.5157	0.0099
Spiromastix	*Chrysosporium*	0.7599	0.0099
Spiromastix	*Enterocarpus*	-0.6172	0.0099
Spiromastix	*Fusarium*	-0.5423	0.0099
Spiromastix	*Lasiobolidium*	-0.5251	0.0099
Spiromastix	*Minimedusa*	-0.5969	0.0099
Spiromastix	*Psathyrella*	0.5421	0.0099
Spiromastix	*Slopeiomyces*	-0.8402	0.0099
Spiromastix	*Solicoccozyma*	-0.5835	0.0099
Spiromastix	*Spizellomyces*	0.5924	0.0099
Spiromastix	*Thelonectria*	0.588	0.0099
Spizellomyces	*Chrysosporium*	0.6206	0.0099
Spizellomyces	*Slopeiomyces*	-0.6129	0.0099
Spizellomyces	*Spiromastix*	0.5924	0.0099
Thelonectria	*Spiromastix*	0.588	0.0099
Thermoascus	*Nigrosabulum*	0.6	0.0099
Trichoderma	*Psathyrella*	0.5924	0.0099
Unknown	*Solicoccozyma*	0.5399	0.0099
Polyscytalum	*Aporpium*	0.5732	0.0099
Polyscytalum	*Auricularia*	0.6131	0.0099
Polyscytalum	*Chrysosporium*	-0.5673	0.0099
Polyscytalum	*Cirrenalia*	0.5706	0.0099
Polyscytalum	*Enterocarpus*	0.5502	0.0099
Polyscytalum	*Golubevia*	-0.5758	0.0099
Polyscytalum	*Moesziomyces*	-0.5165	0.0099
Polyscytalum	*Neocosmospora*	0.6054	0.0099
Polyscytalum	*Slopeiomyces*	0.8008	0.0099
Polyscytalum	*Spiromastix*	-0.834	0.0099
Rhizophydium	*Enterocarpus*	-0.5521	0.0099
Rhizophydium	*Spiromastix*	0.5392	0.0099
Slopeiomyces	*Aporpium*	0.5956	0.0099
Slopeiomyces	*Auricularia*	0.6751	0.0099
Slopeiomyces	*Chrysosporium*	-0.5718	0.0099
Slopeiomyces	*Cirrenalia*	0.648	0.0099
Slopeiomyces	*Enterocarpus*	0.6156	0.0099
Slopeiomyces	*Golubevia*	-0.5766	0.0099
Slopeiomyces	*Moesziomyces*	-0.5564	0.0099
Slopeiomyces	*Neocosmospora*	0.7164	0.0099
Slopeiomyces	*Polyscytalum*	0.8008	0.0099
Slopeiomyces	*Spiromastix*	-0.8649	0.0099
Slopeiomyces	*Unknown*	0.5563	0.0099
Sodiomyces	*Spiromastix*	-0.5354	0.0099
Solicoccozyma	*Spencerozyma*	-0.8005	0.0099
Solicoccozyma	*Spiromastix*	-0.5319	0.0099
Spencerozyma	*Solicoccozyma*	-0.8005	0.0099
Spiromastix	*Aporpium*	-0.7376	0.0099
Spiromastix	*Auricularia*	-0.7853	0.0099
Spiromastix	*Chrysosporium*	0.6547	0.0099
Spiromastix	*Cirrenalia*	-0.6191	0.0099
Spiromastix	*Enterocarpus*	-0.5635	0.0099
Spiromastix	*Golubevia*	0.6808	0.0099
Spiromastix	*Moesziomyces*	0.6601	0.0099
Spiromastix	*Neocosmospora*	-0.7184	0.0099
Spiromastix	*Polyscytalum*	-0.834	0.0099
Neocosmospora	*Cirrenalia*	-0.6248	0.0099
Neocosmospora	*Psathyrella*	0.6049	0.0099
Penicillium	*Polyscytalum*	-0.5458	0.0099
Penicillium	*Spencerozyma*	0.5152	0.0099
Phomatospora	*Monosporascus*	0.5714	0.0099
Plectosphaerella	*Ceratobasidium*	-0.5808	0.0099
Plectosphaerella	*Spencerozyma*	0.5703	0.0099
Plectosphaerella	*Spiromastix*	0.6269	0.0099
Poaceascoma	*Lasiobolidium*	-0.5526	0.0099
Poaceascoma	*Spencerozyma*	0.7259	0.0099
Poaceascoma	*Spiromastix*	0.7905	0.0099
Polyscytalum	*Aporpium*	0.695	0.0099
Polyscytalum	*Auricularia*	0.5524	0.0099
Polyscytalum	*Emericellopsis*	0.5661	0.0099
Polyscytalum	*Funneliformis*	0.5772	0.0099
Polyscytalum	*Fusarium*	-0.533	0.0099
Polyscytalum	*Penicillium*	-0.5458	0.0099
Psathyrella	*Aspergillus*	0.6552	0.0099
Psathyrella	*Cirrenalia*	-0.5547	0.0099
Psathyrella	*Dissophora*	0.6333	0.0099
Psathyrella	*Malassezia*	-0.5497	0.0099
Psathyrella	*Minimedusa*	0.7953	0.0099
Psathyrella	*Neocosmospora*	0.6049	0.0099
Psathyrella	*Thelonectria*	0.5504	0.0099
Sarocladium	*Spencerozyma*	0.5688	0.0099
Spencerozyma	*Penicillium*	0.5152	0.0099
Spencerozyma	*Plectosphaerella*	0.5703	0.0099
Spencerozyma	*Poaceascoma*	0.7259	0.0099
Spencerozyma	*Sarocladium*	0.5688	0.0099
Spencerozyma	*Spiromastix*	0.8837	0.0099
Spiromastix	*Plectosphaerella*	0.6269	0.0099
Spiromastix	*Poaceascoma*	0.7905	0.0099
Spiromastix	*Spencerozyma*	0.8837	0.0099
Thelonectria	*Aspergillus*	0.7568	0.0099
Thelonectria	*Auricularia*	-0.6497	0.0099
Thelonectria	*Dactylonectria*	0.5102	0.0099

				Spiromastix	*Rhizophydium*	0.5392	0.0099	*Thelonectria*	*Emericellopsis*	-0.5674	0.0099
				Spiromastix	*Slopeiomyces*	-0.8649	0.0099	*Thelonectria*	*Funneliformis*	-0.539	0.0099
				Spiromastix	*Sodiomyces*	-0.5354	0.0099	*Thelonectria*	*Fusarium*	0.912	0.0099
				Spiromastix	*Solicoccozyma*	-0.5319	0.0099	*Thelonectria*	*Psathyrella*	0.5504	0.0099
				Spiromastix	*Unknown*	-0.5216	0.0099	*Thelonectria*	*Verrucostoma*	0.6075	0.0099
				Spizellomyces	*Golubevia*	0.5278	0.0099	*Trichoderma*	*Dactylonectria*	0.511	0.0099
				Spizellomyces	*Moesziomyces*	0.5191	0.0099	*Verrucostoma*	*Fusarium*	0.5282	0.0099
				Unknown	*Auricularia*	0.557	0.0099	*Verrucostoma*	*Thelonectria*	0.6075	0.0099
				Unknown	*Cirrenalia*	0.5029	0.0099				
				Unknown	*Slopeiomyces*	0.5563	0.0099				
				Unknown	*Spiromastix*	-0.5216	0.0099				
				Verrucostoma	*Microthecium*	0.569	0.0099				

Table S6. Relative proportion (%) of fungal function from soil-plant compartments at each irrigation regime inferred by FunGuild.

	Bulk soil			Rhizosphere			Root		
	SWD[a]	MWD[b]	AWD[c]	SWD	MWD	AWD	SWD	MWD	AWD
Pathotroph	24.52±0.67 Ba	26.13±1.35 Ba	23.62±3.08 Ba	27.79±3.22 Ba	24.20±0.98 Ba	23.97±1.03 Ba	26.25±3.29 Bb	34.50±0.92 Ba	28.41±0.52 Bab
Saprotroph	44.96±1.97 Aa	42.27±0.52 Aa	46.50±2.57 Aa	45.63±1.91 Aa	45.16±2.87 Aa	47.87±2.42Aa	52.28±5.07 Aa	41.51±1.34 Aa	45.22±2.14 Aa
Symbiotroph	25.37±1.94 Ba	24.50±0.73 Ba	22.11±1.49 Ba	22.21±2.57 Ba	23.38±3.07 Ba	20.32±2.12 Ba	17.48±2.23 BCa	20.17±0.51 Ca	16.98±2.14 Ca
Unassigned	5.16±0.74 Ca	7.10±1.06 Ca	7.77±1.91 Ca	4.37±0.31 Cb	7.26±1.22 Cab	7.85±0.74 Ca	3.98±0.37 Ca	3.81±0.39 Da	9.38±1.60 Ca

[a] Severe Water Deficit

[b] Moderate Water Deficit

[c] Absence of Water Deficit

Tukey's test at $P < 0.05$ level. Means followed by the same letter do not differ significantly ($P < 0.05$). Capital letters are for comparison of means among functional groups within each irrigation regime. Small letters are for comparison of means among irrigation regimes within each functional group.

Table S7. Compositions and relative abundance (%) of fungal functional groups (guild) inferred by FunGuild.

		Bulk soil			Rhizosphere			Root		
		**SWD**[a]	**MDW**[b]	**AWD**[c]	**SWD**	**MWD**	**AWD**	**SWD**	**MWD**	**AWD**
Pathotroph	Plant Pathogen	8.02 Aa	10.21 Aa	7.57 Aa	15.79 Aa	15.28 Aa	12.77 Aa	17.49 Aab	20.27 Aa	11.94 Ab
	Animal Pathogen	6.81 Aa	6.37 ABa	7.49 Aa	4.44 Ba	4.21 Ba	5.72 Ba	3.80 Ba	5.71 Ba	7.90 Ba
	Fungal Parasite	3.34 Ba	3.59 Ba	3.49 Aa	2.32 Ba	0.82 Ca	1.07 Ca	1.58 Ba	3.99 Ba	3.32 Da
	Lichen Parasite	6.35 Aa	5.95 ABa	5.07 Aa	5.25 Ba	3.89 Ba	4.42 Ba	3.39 Ba	4.54 Ba	5.24 Ca
Saprotroph	Soil Saprotroph	13.56 Ba	10.32 Ba	12.15 Ba	11.21 Ba	8.70 Ba	9.80 Ba	4.23 Ba	6.10 Ba	8.50 Ba
	Wood Saprotroph	7.41 Ca	8.82 Ba	8.50 Ba	7.42 BCa	5.34 BCb	10.16 Bab	10.93 ABa	8.63 Ba	10.22 Ba
	Dung Saprotroph	2.28 Da	2.90 Da	1.43 Da	3.27 Ca	4.60 BCa	3.63 Ca	6.15 Ba	1.56 Cb	3.14 Cab
	Plant Saprotroph	2.37 Db	2.62 Ca	3.77 Ca	3.29 Ca	1.86 Ca	2.64 Ca	3.01 Ba	6.44 Ba	5.04 Ca
	Undefined Saprotroph	19.34 Aa	17.60 Aa	20.65 Aa	20.44 Aa	24.66 Aa	21.64 Aa	27.97 Aa	18.77 Aa	18.33 Aa
Symbiotroph	Endophyte	21.24 Aa	19.44 Aa	20.68 Aa	16.31 Aa	14.82 Aa	16.17 Aa	7.74 Aa	9.79 Aa	15.25 Aa
	Arbuscular Mycorrhizal	4.12 Ba	5.05 Ba	1.43 Ba	5.89 Ba	8.56 Ba	4.15 Ba	9.74 Aa	10.38 Aa	1.74 Ab
Unassigned		5.16 a	7.10 a	7.77 a	4.37 b	7.26 ab	7.85 a	3.99 a	3.82 a	9.38 a

[a] Severe Water Deficit

[b] Moderate Water Deficit

[c] Absence of Water Deficit

Tukey's test at $P < 0.05$ level. Means followed by the same letter do not differ significantly ($P< 0.05$). Capital letters are for comparisons of means of functional groups within each trophic mode, irrigation regime and soil-plant compartment. Small letters are for comparison of means of each functional group among irrigation regimes within each soil-plant compartment.

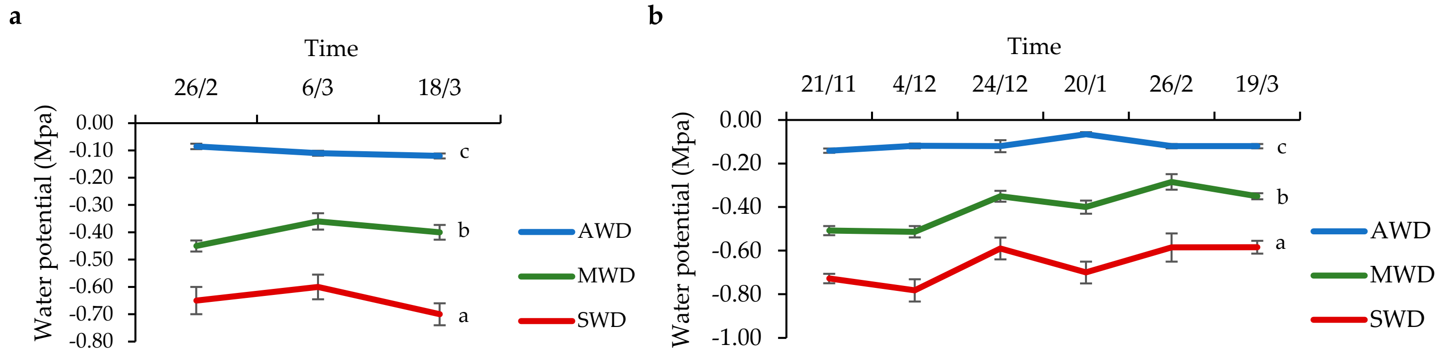

Figure S1. Predawn leaf water potential at the irrigation regimes. Absence of water deficit (AWD), moderate water deficit (MWD) and severe water deficit (SWD). (a) Growing season 2018-2019 and (b) 2019-2020. Tukey´s test at *p*-value < 0.05 level. Means followed by the same letter do not differ significantly.

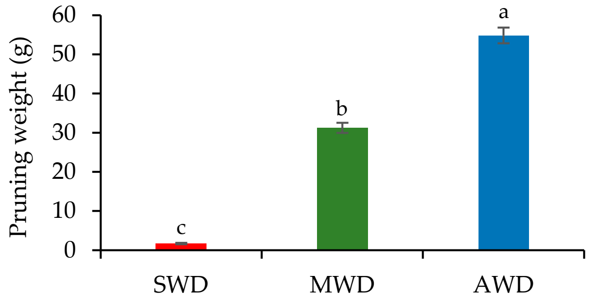

Figure S2. Pruning weight at the irrigation regimes. Absence of water deficit (AWD), moderate water deficit (MWD) and severe water deficit (SWD). Pruning made in winter 2020. Tukey´s test at *p*-value < 0.05 level. Means followed by the same letter do not differ significantly.

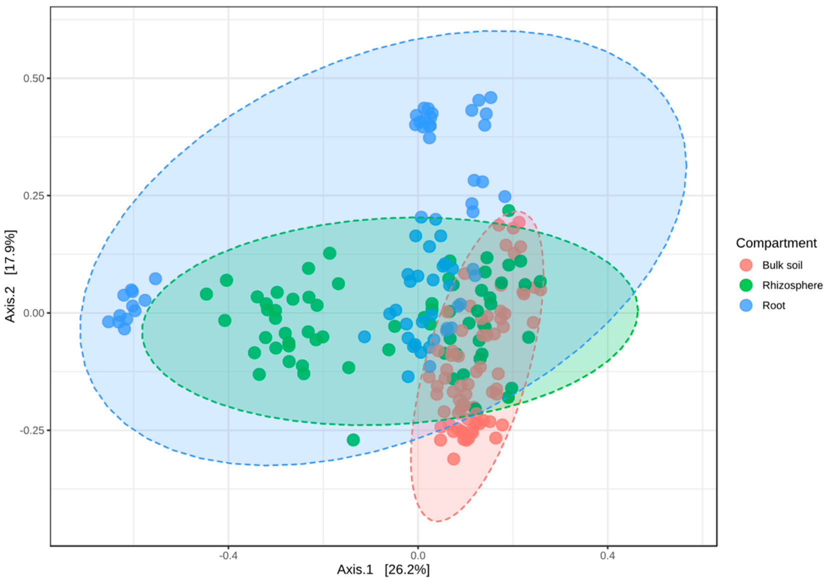

Figure S3. Principal Coordinate Analysis (PCoA) based on Bray Curtis dissimilarity metrics, showing the distance in the fungal communities among soil-plant compartments.

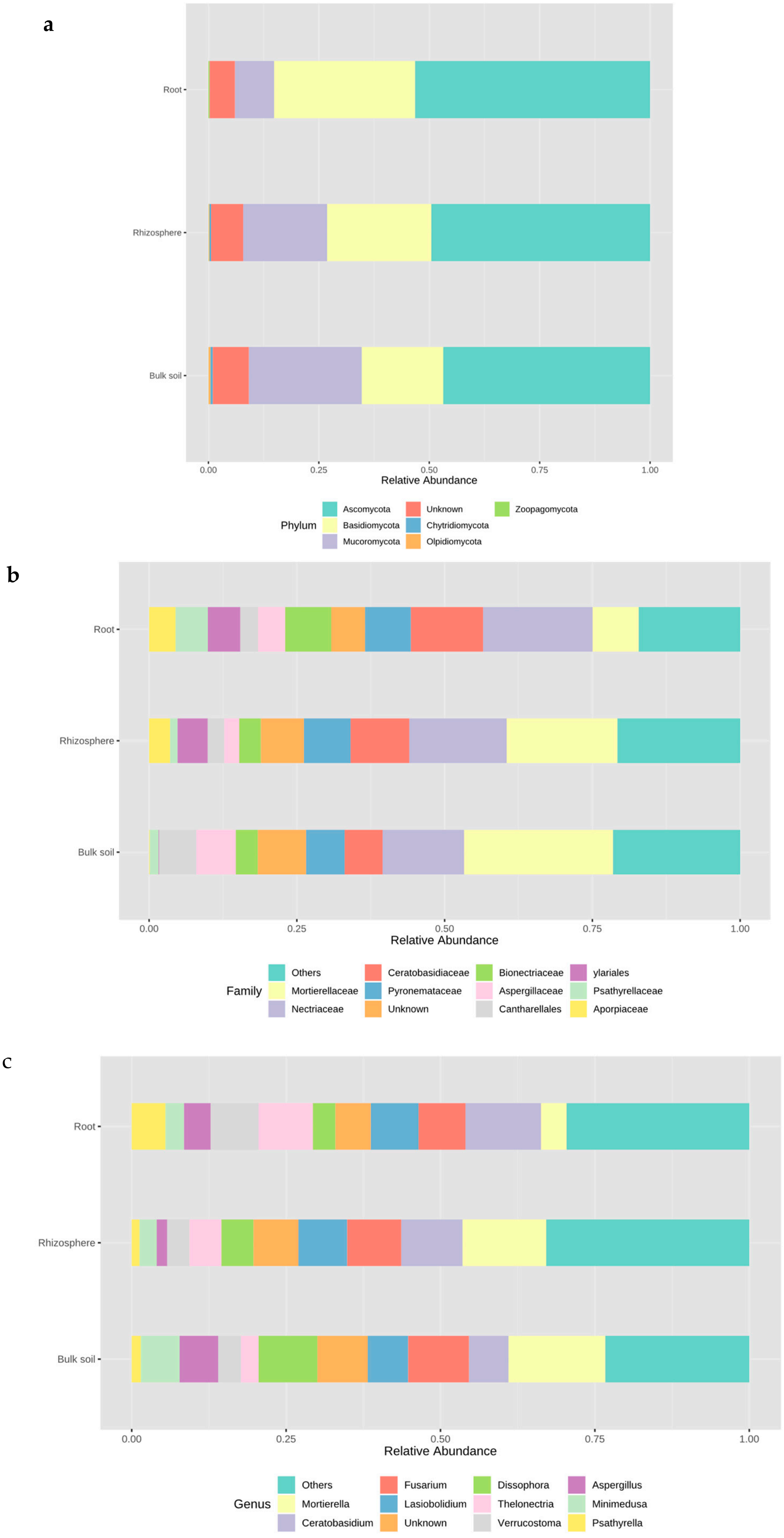

Figure S4. Relative abundance of the most abundant (a) phyla, (b) families and (c) genera in bulk-soil, rhizosphere and root samples

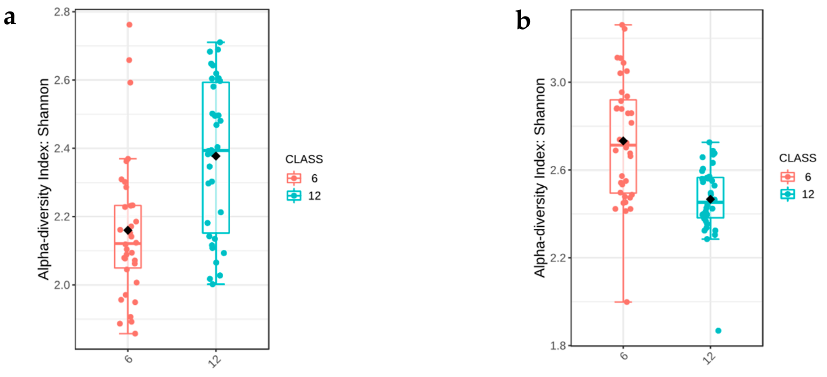

Figure S5. Boxplot illustrating the differences in Shannon diversity measure of the fungal communities in (a) root and (b) bulk soil between 6- and 12-month sampling time.

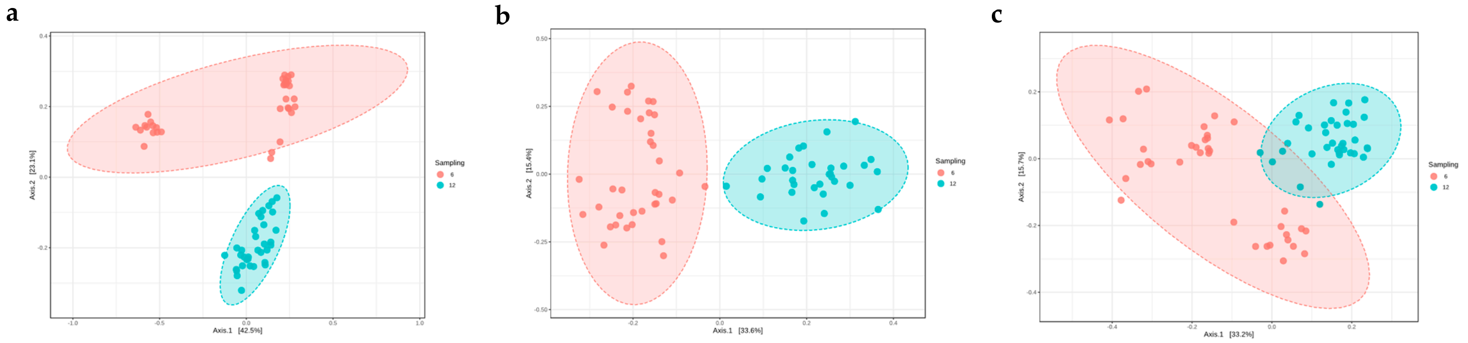

Figure S6. Principal Coordinate Analysis (PCoA) based on Bray Curtis dissimilarity metrics, showing the distance in the fungal communities in (a) root, (b) rhizosphere and (c) bulk soil between 6- and 12-month sampling time.

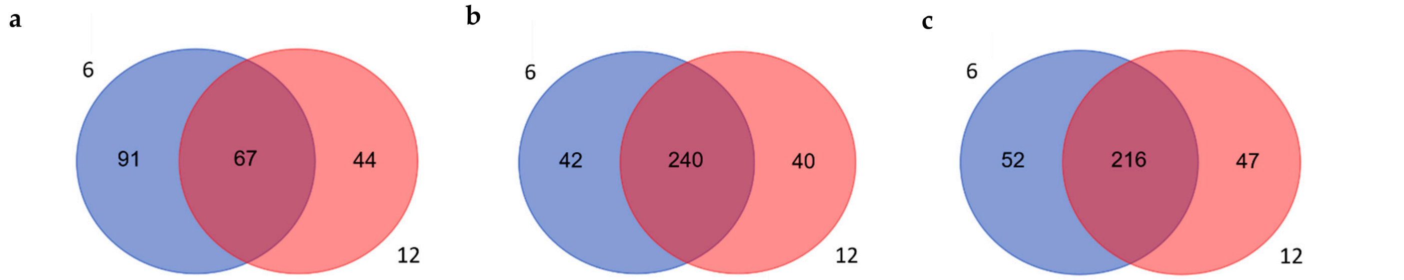

Figure S7. Venn diagram illustrating the overlap of the OTUs identified in the fungal microbiota between 6- and 12-month sampling time in the (a) root, (b) rhizosphere and (c) bulk-soil.

a

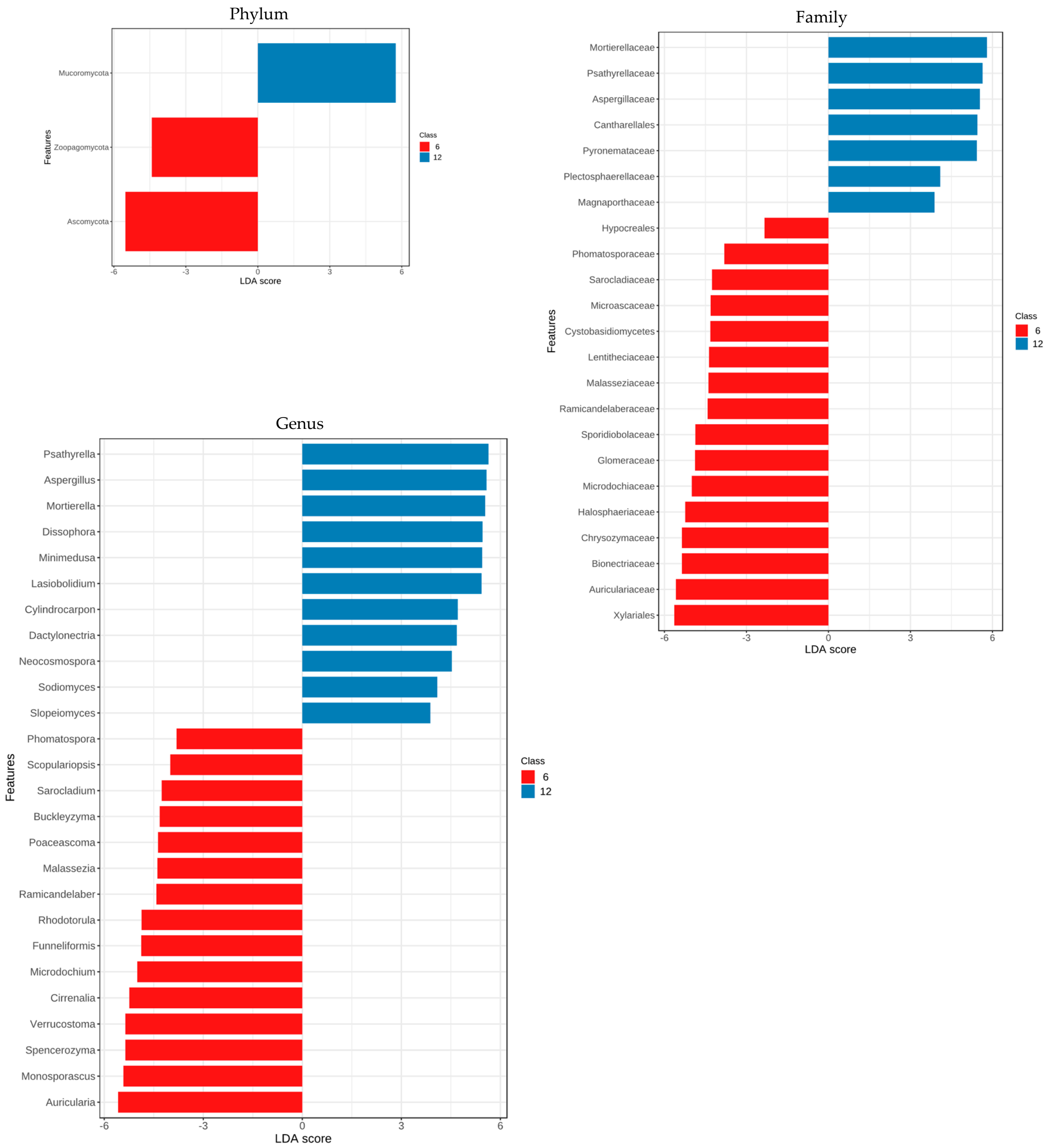
Phylum
Mucoromycota
Zoopagomycota
Ascomycota
Features
LDA score
-6
-3
0
3
6
Class
6
12
Family
Mortierellaceae
Psathyrellaceae
Aspergillaceae
Cantharellales
Pyronemataceae
Plectosphaerellaceae
Magnaporthaceae
Hypocreales
Phomatosporaceae
Sarocladiaceae
Microascaceae
Cystobasidiomycetes
Lentitheciaceae
Malasseziaceae
Ramicandelaberaceae
Sporidiobolaceae
Glomeraceae
Microdochiaceae
Halosphaeriaceae
Chrysozymaceae
Bionectriaceae
Auriculariaceae
Xylariales
Features
LDA score
Class
6
12
Genus
Psathyrella
Aspergillus
Mortierella
Dissophora
Minimedusa
Lasiobolidium
Cylindrocarpon
Dactylonectria
Neocosmospora
Sodiomyces
Slopeiomyces
Phomatospora
Scopulariopsis
Sarocladium
Buckleyzyma
Poaceascoma
Malassezia
Ramicandelaber
Rhodotorula
Funneliformis
Microdochium
Cirrenalia
Verrucostoma
Spencerozyma
Monosporascus
Auricularia
Features
LDA score
Class
6
12

b

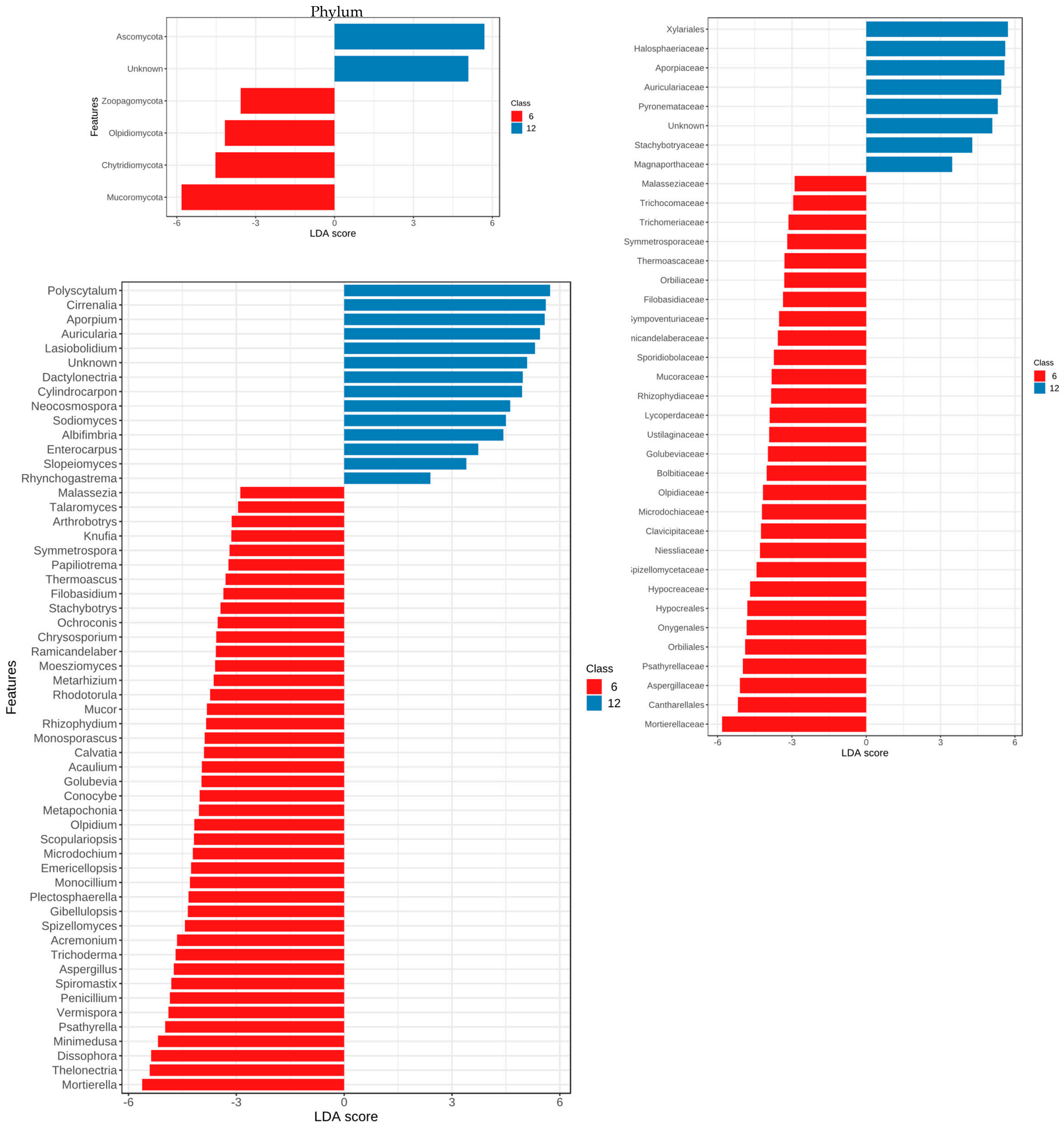

Phylum
Ascomycota
Unknown
Zoopagomycota
Olpidiomycota
Chytridiomycota
Mucoromycota
Features
LDA score
-6
-3
0
3
6
Class
6
12
Polyscytalum
Cirrenalia
Aporpium
Auricularia
Lasiobolidium
Unknown
Dactylonectria
Cylindrocarpon
Neocosmospora
Sodiomyces
Albifimbria
Enterocarpus
Slopeiomyces
Rhynchogastrema
Malassezia
Talaromyces
Arthrobotrys
Knufia
Symmetrospora
Papiliotrema
Thermoascus
Filobasidium
Stachybotrys
Ochroconis
Chrysosporium
Ramicandelaber
Moesziomyces
Metarhizium
Rhodotorula
Mucor
Rhizophydium
Monosporascus
Calvatia
Acaulium
Golubevia
Conocybe
Metapochonia
Olpidium
Scopulariopsis
Microdochium
Emericellopsis
Monocillium
Plectosphaerella
Gibellulopsis
Spizellomyces
Acremonium
Trichoderma
Aspergillus
Spiromastix
Penicillium
Vermispora
Psathyrella
Minimedusa
Dissophora
Thelonectria
Mortierella
Features
LDA score
Class
6
12
Xylariales
Halosphaeriaceae
Aporpiaceae
Auriculariaceae
Pyronemataceae
Unknown
Stachybotryaceae
Magnaporthaceae
Malasseziaceae
Trichocomaceae
Trichomeriaceae
Symmetrosporaceae
Thermoascaceae
Orbiliaceae
Filobasidiaceae
ympoventuriaceae
nicandelaberaceae
Sporidiobolaceae
Mucoraceae
Rhizophydiaceae
Lycoperdaceae
Ustilaginaceae
Golubeviaceae
Bolbitiaceae
Olpidiaceae
Microdochiaceae
Clavicipitaceae
Niessliaceae
pizellomycetaceae
Hypocreaceae
Hypocreales
Onygenales
Orbiliales
Psathyrellaceae
Aspergillaceae
Cantharellales
Mortierellaceae
LDA score
Class
6
12
